# Restraining the escape of lattice oxygen enables superior cyclic performance towards high-voltage Ni-rich cathodes

**DOI:** 10.1093/nsr/nwac166

**Published:** 2022-08-18

**Authors:** Haifeng Yu, Huawei Zhu, Hongliang Jiang, Xiaozhi Su, Yanjie Hu, Hao Jiang, Chunzhong Li

**Affiliations:** Key Laboratory for Ultrafine Materials of Ministry of Education, School of Materials Science and Engineering, East China University of Science and Technology, Shanghai 200237, China; Key Laboratory for Ultrafine Materials of Ministry of Education, School of Materials Science and Engineering, East China University of Science and Technology, Shanghai 200237, China; Shanghai Engineering Research Center of Hierarchical Nanomaterials, School of Chemical Engineering, East China University of Science and Technology, Shanghai 200237, China; Shanghai Synchrotron Radiation Facility, Shanghai Advanced Research Institute, Chinese Academy of Sciences, Shanghai 201210, China; Key Laboratory for Ultrafine Materials of Ministry of Education, School of Materials Science and Engineering, East China University of Science and Technology, Shanghai 200237, China; Key Laboratory for Ultrafine Materials of Ministry of Education, School of Materials Science and Engineering, East China University of Science and Technology, Shanghai 200237, China; Shanghai Engineering Research Center of Hierarchical Nanomaterials, School of Chemical Engineering, East China University of Science and Technology, Shanghai 200237, China; Key Laboratory for Ultrafine Materials of Ministry of Education, School of Materials Science and Engineering, East China University of Science and Technology, Shanghai 200237, China; Shanghai Engineering Research Center of Hierarchical Nanomaterials, School of Chemical Engineering, East China University of Science and Technology, Shanghai 200237, China

**Keywords:** Ni-rich cathode, lattice oxygen, dual modification, long cycle life, Li-ion battery

## Abstract

Layered Ni-rich cathodes, operating at high voltage with superior cyclic performance, are required to develop future high-energy Li-ion batteries. However, the worst lattice oxygen escape at the high-voltage region easily causes structural instability, rapid capacity fading and safety issues upon cycling. Here, we report a dual-track strategy to fully restrain the escape of lattice oxygen from Ni-rich cathodes within 2.7–4.5 V by one-step Ta doping and CeO_2_ coating according to their different diffusion energy barriers. The doped Ta can alleviate the charge compensation of oxygen anions as a positive charge centre to reduce the lattice oxygen escape and induce the formation of elongated primary particles, significantly inhibiting microcrack generation and propagation. Additionally, the layer of CeO_2_ coating effectively captures the remaining escaped oxygen and then the captured oxygen feeds back into the lattice during subsequent discharge. The resultant Ni-rich cathode enables a capacity of 231.3 mAh g^−1^ with a high initial coulombic efficiency of 93.5%. A pouch-type full cell comprising this cathode and a graphite anode exhibits >1000 times life cycles at 1C in the 2.7–4.5 V range, with 90.9% capacity retention.

## Teaser

The breathable lattice oxygen in the cathode materials enables high-performance and safety lithium-ion batteries.

## INTRODUCTION

Layered oxide cathodes (LiNi_x_Co_y_Mn_1−x−y_O_2_), such as LiNi_0.8_Co_0.1_Mn_0.1_O_2_ (NCM811), demonstrate great academic and industrial merits in advanced lithium-ion batteries (LIBs) owing to their high energy density and acceptable cost [1–3]. The present state-of-the-art NCM811//Si/C or NCM811//Li anode cell can deliver a top energy density of ∼350 Wh kg^−1^ [4–6]. To increase the energy density up to 400 Wh kg^−1^ and beyond, further increasing the Ni content and broadening the work voltage to ≥4.5 V in layered oxide cathodes is necessary [7,8]. Viewing from the crystal structure of Ni-based layered cathodes, the alternating occupation of oxygen octahedral interstices by Li/Ni ions along the [001] direction causes σ-type hybridization between the Ni^3+^/Ni^4+^: e_g_ electron orbital and the O^2−^: 2p orbital [9,10]. As for high-Ni oxide (Ni-rich) cathodes, when charging to 4.1 V, electron loss in O^2−^: 2p orbitals begins to happen, with d-hole generation in view of the rapid Ni^3+^ oxidation [11,12]. When the voltage is increased to >4.3 V, additional oxygen anions participate in the charge compensation to balance the as-formed Ni^4+^ due to >80% Li^+^ extraction [13]. This disadvantage weakens the electrostatic repulsion between oxygen layers with a significant contraction of lattice parameters (H2–H3 phase transition) [14]. Moreover, the continuous charge loss of lattice oxygen expedites the escape of oxygen anions [15]. The resultant oxygen free radicals easily give rise to electrolyte decomposition with undesirable side reactions [16]. These weaknesses pose threats to capacity retention and create thermal issues during prolonged cycling under high operation voltage. Therefore, retaining the lattice oxygen of Ni-rich cathodes without loss, even charging to >4.3 V to achieve higher-energy-density LIBs, is a huge challenge.

With this perspective, the type, valence and coordination mode of transition metal (TM) ions in their oxides can significantly affect electron arrangement and orbital hybridization [17,18], providing the possibility of regulating lattice oxygen for layered oxide cathodes. Previous studies unveiled that lattice oxygen is stabilized mainly by decreasing σ-type orbital hybridization and hence restraining the oxygen anion charge compensation caused by the valence change of transitional metal ions [19,20]. However, an excessive change in composition damages order-layered oxide cathodes (LiNiO_2_ and LiCoO_2_), greatly sacrificing the specific capacity with sluggish delithiation–lithiation dynamics. Recently, substantial studies have focused on decreasing the irreversible lattice oxygen without destroying bulk structure by doping various high bonding energy elements, such as Ti, W and B [21,22]. Unfortunately, a small quantity of lattice oxygen loss still occurs, which cannot be ignored in Ni-rich cathodes, especially when operating in high-voltage regions and long-term cycling. The stress corrosion cracking is becoming worse, ultimately leading to severe structure degradation and surface side effects and thus deteriorating durability [23,24].

Herein, a dual-track strategy has been deployed to fully avoid the lattice oxygen escape of Ni-rich cathodes when operated at 2.7–4.5 V by combining Ta doping and CeO_2_ coating in one step based on their remarkable differences in diffusion energy barriers. Ta doping not only can greatly stabilize the lattice oxygen due to the strong Ta–O bond but also can help form the elongated primary particles for inhibiting microcrack generation and propagation. Importantly, the CeO_2_ coating layer can effectively capture the remaining escaped oxygen and then feedback to lattice during following discharge. The dual-track synergy allows the modified cathode to possess a high initial coulombic efficiency (ICE) of 93.5% and retain 90.9% of its initial capacity after 1000 cycles at 2.7–4.5 V in a pouch-type full cell.

## RESULTS AND DISCUSSION

**Figure 1.**
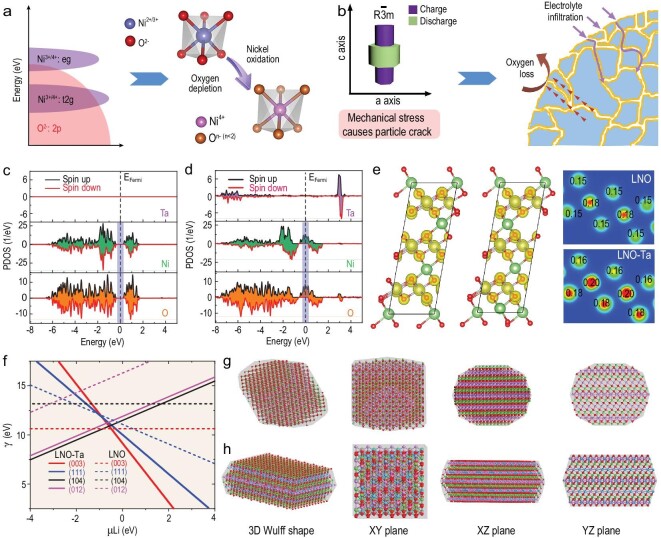
Escape mechanism and stabilization of lattice oxygen in a Ni-rich cathode. (a) Schematic of oxygen depletion caused by the orbital hybridization of Ni^3+^/Ni^4+^: e_g_–O^2−^: 2p during Ni oxidation. (b) Illustration of oxygen loss aggravated by the side effects from the anisotropic lattice deformation enabling electrolyte infiltration during delithiation or lithiation. (c and d) Calculated PDOS of Ni: 3d, O: 2p and Ta: 5d of (111) planes. (e) 3D and 2D contour maps of charge-density distribution for LiNiO_2_ (LNO) and Ta-doped LNO (LNO–Ta) after removing 90% Li-ion. (f) Surface energy of (003), (111), (104) and (012) planes as a function of chemical potential of Li (μLi). (g and h) Corresponding equilibrium shapes for LNO and LNO–Ta.

The lattice oxygen escape in Ni-rich layered cathodes is mainly caused by the following two points. One is that partial oxygen anions are inevitably oxidized to O^−^ radicals or O_2_ gas at a high state of charge (SOC) due to the aforementioned σ-type hybridization (Fig. 1a). The other is that oxygen anions are further consumed because of the side effects from the electrolyte infiltration along intergranular crack enabling by the anisotropic volume change (Fig. 1b). Considering that Ta^5+^ and Ni^3+^ have similar ionic radii and Ta–O has a high bonding energy of 839 kJ mol^−1^ [25], density function theory (DFT) calculations have been conducted to investigate the effects of Ta doping on the electronic structure and surface energy of Ni-rich cathodes. We first give the projected density of states (PDOS) of O: 2p, Ni: 3d and Ta: 5d on the basis of the (111) planes (Fig. 1c and d) and other planes (Supplementary Fig. S1) of LiNiO_2_ and Ta-doped LiNiO_2_ after removing 90% Li-ion. Although the obvious orbital overlapping of Ni: 3d and O: 2p is observed for the two samples, the unoccupied O: 2p orbitals above the Fermi level are significantly reduced for Ta-doped LiNiO_2_, indicating that Ta doping can effectively alleviate the charge depletion of lattice oxygen [21]. Additionally, Ta doping significantly changes the orbital characteristics. For (111) planes, the asymmetrical orbitals across the Fermi level appear in the PDOS of Ni: 3d and O: 2p for Ta-doped LiNiO_2_. This phenomenon suggests that doped Ta^5+^ ions, as the positive charge centre, provide extra electrons to the neighbouring Ni and O ions, thereby causing the shift of the Ni: 3d–O: 2p hybrid orbital to a high energy level. The hybrid orbital is far away from the O: 2p orbital, which is beneficial in alleviating the charge compensation of O anions when the charge loss occurs in Ni ions. The charge-density distribution shown in Fig. 1e and Supplementary Fig. S2 also exhibits a mass of electron deficiency at Ta sites. The corresponding 2D maps of charge-density distribution further indicate an increasing charge density of Ni and O ions around Ta. Consequently, Ta doping stabilizes the lattice oxygen of Ni-rich cathodes. Meanwhile, the surface energies of different crystal planes are further calculated to investigate the effects of Ta doping on the crystal growth of primary particles. Thermodynamically, the morphology of layered LiTMO_2_ is mainly determined by (003), (111), (104) and (012) planes [26]. Therefore, these four planes of LiNiO_2_ and Ta-doped LiNiO_2_ are selected (see the detailed crystal structures in Supplementary Fig. S3) and the linear functions of surface energy and chemical potential (μLi) are established (Fig. 1f). Note that the (003) and (111) surface energies of Ta-doped LiNiO_2_ remarkably decrease as chemical potential increases compared with those of LiNiO_2_. In view of the Li-rich surface of a Ni-rich cathode (i.e. high chemical potential), these two planes dominate the crystal shape. According to the principles of crystal symmetry and minimized total surface energy, the equilibrium morphologies of LiNiO_2_ (Fig. 1g) and Ta-doped LiNiO_2_ (Fig. 1h), called the Wulff morphologies [27], are simulated on the basis of the calculated surface energy of various crystal planes (Supplementary Table S1). The Ta-doped LiNiO_2_ exhibits a flat-plate shape with obvious orientation, whereas the approximate equiaxed cube-like shape is obtained for LiNiO_2_. The microsphere, aggregated by oriented primary particles, effectively dissipates internal mechanical stress change and significantly reduces parasitic reactions during delithiation or lithiation [28].

**Figure 2.**
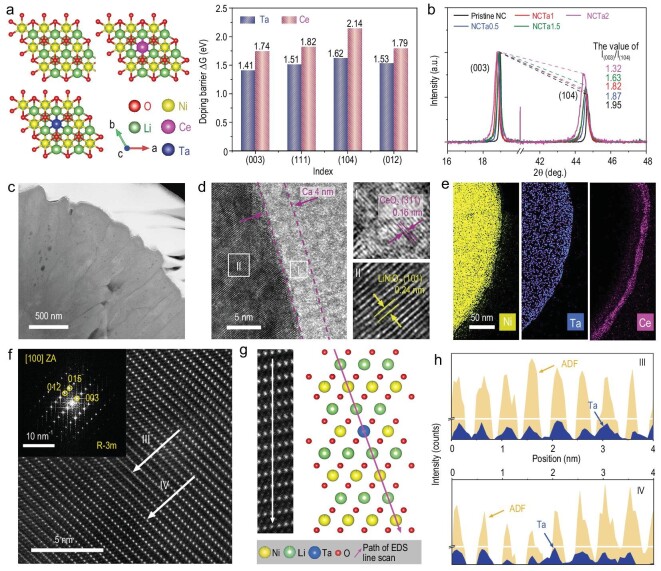
Theoretical analysis and structural characterization. (a) Diagrams of LiNiO_2_, Ta-doped LiNiO_2_ and Ce-doped LiNiO_2_ and their doping energy barriers on (003), (111), (104) and (012) planes. (b) Intensity ratios of (003)/(104) XRD peaks of LiNi_0.9_Co_0.1_O_2_ with various Ta contents after normalizing the (003) peak. (c) STEM–HAADF image of the cross section for NCTa1–CeO_2_. (d) High-resolution TEM. (e) Corresponding EDS mapping images of NCTa1–CeO_2_. (f) Cs-STEM–HAADF image with the corresponding FT image of NCTa1–CeO_2_. (g) Enlarged STEM–HAADF image and the diagram of EDS line scan. (h) EDS line analysis of annular dark-field signals and Ta signals.

Although doping heterogeneous elements can enhance the stability of the lattice oxygen of Ni-rich cathodes, a non-negligible loss of lattice oxygen still occurs, especially at high work voltage (e.g. ≥4.5 V). A proper coating layer is indispensable. The CeO_2_ is a typical oxygen buffer, which is helpful to avoid the loss of residual unstable lattice oxygen [29]. It is expected to concurrently achieve efficient Ta doping and uniform CeO_2_ coating from the angle of practice applications. The theoretical calculation demonstrates that the doping barrier energies of Ta are much lower than those of Ce in various crystal planes of Ni-rich cathodes (Fig. 2a and Supplementary Fig. S4). Ce^4+^ ions have a larger ionic radius of 1.07 Å than Ni^3+^ ions (0.56 Å). These features imply that Ce ions are difficult to dope into the lattice. Here, a pre-coating and post-annealing strategy is applied to obtain Ta-doped and CeO_2_-coated Ni-rich cathodes, as shown in Supplementary Fig. S5. Ta-doping optimization is first performed. Figure 2b illustrates the intensity ratio of the (003)/(104) XRD peaks of Ta-doped LiNi_0.9_Co_0.1_O_2_ cathodes with different Ta contents after normalizing the (003) peak. The intensity ratios gradually reduce as Ta contents improve, implying the increase in Li/Ni disorder [30]. Associating with the corresponding XPS spectra (Supplementary Fig. S6), ICP–AES (Supplementary Table S2) and XRD Rietveld refinements (Supplementary Figs S7 and S8 and Supplementary Table S3), the 1% Ta-doped LiNi_0.9_Co_0.1_O_2_ (NCTa1) displays the largest lattice parameters in terms of *a*-axis, *c*-axis and unit volume, and the appropriate Li/Ni disorder. Supplementary Fig. S9 shows the morphologies of the pristine and Ta/Ce surface-enriched Ni_0.9_Co_0.1_(OH)_2_ precursors. After lithiation, Ta doping and CeO_2_ coating are achieved (NCTa1–CeO_2_). The CeO_2_ weight ratio is ∼0.51% (Supplementary Fig. S10), which is consistent with the feed ratios. The lattice parameters have no obvious change between NCTa1 and NCTa-CeO_2_ (Supplementary Table S3), indicating that Ce ions only stay on the surface to form a CeO_2_ coating layer. The modification process has no obvious effects on the morphologies of spherical secondary particles (Supplementary Fig. S11).

Notably, the sizes of primary particles notably decrease after Ta doping, which matches well with the peak broadening of (003) in XRD patterns (Supplementary Fig. S7) and the aforementioned DFT result (Fig. 1f). The cross-section SEM images in Supplementary Fig. S12 verify that NCTa1–CeO_2_ is composed of radially oriented primary particles with a higher length–diameter ratio than pristine Ni-rich oxides (pristine NC). Additionally, the atomic ratios of Ta ions are always ∼1% from the surface to the centre of secondary particles (Supplementary Fig. S13), indicating the uniform Ta element distributions. The scanning transmission electron microscopy (STEM) image of NCTa1–CeO_2_ is also provided in Fig. 2c, exhibiting a close arrangement. The further magnification of STEM image discloses a uniform CeO_2_ coating layer with a thickness of ∼4.0 nm, as shown in Fig. 2d. The lattice spacing of 0.16 nm (region I) is indexed to the (311) plane of the CeO_2_ (JCPDS 34-0394), whereas that of 0.24 nm (region II) is attributed to the (101) plane of the LiNiO_2_ (JCPDS 09-0063). The corresponding Fourier transform (FT) images are shown in Supplementary Fig. S14. To further highlight the uniform CeO_2_ coating, 2 wt% content is prepared and analysed using a low-magnification TEM image. The corresponding EDS mapping and high-angle annular dark-field (HAADF) images are displayed in Fig. 2e and Supplementary Fig. S15. Ta uniformly distributes on the bulks and Ce only enriches on the surface, further verifying the successful preparation of Ta-doped and CeO_2_-coated Ni-rich cathodes. As for pristine NC, randomly primary particles are aggregated with a clean surface (Supplementary Fig. S16). The double aberration-corrected STEM (Cs-STEM) with EDS accessory is performed to assess the integrity of the crystal structure and ascertain the accurate doping sites of Ta ions in Ni-rich cathodes. The Cs-STEM image (Fig. 2f) acquired in HAADF mode, along with [100] orientation and the corresponding FT image (the inset of Fig. 2f), indicates that Ta doping has no effect on the intrinsic crystal structure. Layers with bright spots can be assigned, and TM layers and dark regions between two TM layers can be recognized as Li layers. The EDS line scanning path has been chosen to pass through the TM and Li sites in turn, as illustrated in Fig. 2g. The results are shown in Fig. 2h. The characteristic signals of Ta only appear in TM sites, indicating that the doped Ta occupies the octahedral interstices in the TM layer of Ni-rich cathodes.

**Figure 3.**
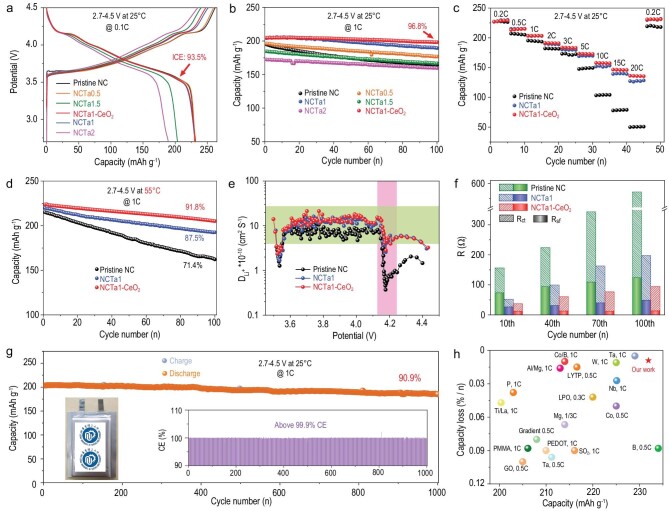
Superior electrochemical performance and cycling stability in the half cell and full cell. (a) Initial charge–discharge curves at 0.1C and (b) cycle performance at 1C within 2.7–4.5 V for all samples. (c) Specific capacities at 0.2–20C, (d) cycle performance at 55°C, (e) Li-ion diffusion coefficient based on GITT data and (f) comparison of *R_sf_* and *R_ct_* at different cycles within 2.7–4.5 V for pristine NC, NCTa1 and NCTa1–CeO_2_. (g) Cycle stability at 1C of NCTa1–CeO_2_//graphite full cells within 2.7–4.5 V. (h) Comparisons of the 0.1C discharge capacity and capacity loss per cycle with the reported Ni-rich cathodes.

The delithiation–lithiation capabilities of all samples are evaluated by assembling coin-type half cells with a charging cut-off voltage as high as 4.5 V. Figure 3a displays their initial charge–discharge curves at 0.1C. NCTa1–CeO_2_ shows the highest ICE of 93.5%. After 100 cycles at 1C, 96.8% capacity retention is achieved for NCTa1–CeO_2_ (Fig. 3b). The CeO_2_ coating notably reduces the voltage hysteresis (ΔV) from 0.052 to 0.025 V (Supplementary Fig. S17). Additionally, 1% Ta doping is optimal as predicted, which exhibits the best electrochemical performance, including ICE, specific capacity and cycling stability (Supplementary Fig. S18). Figure 3c shows the rate capability of pristine NC, NCTa1 and NCTa1–CeO_2_ at 0.2–20°C. NCTa1 and NCTa1–CeO_2_ deliver much higher specific capacities than that of NC at various rates. The values of the three samples can recover weel once the current density reduces back to 0.2C. Notably, NCTa1–CeO_2_ exhibits a specific capacity of 136 mAh g^−1^ at 20C, slightly higher than NCTa1 (127 mAh g^−1^), implying a thin cathode–electrolyte interface (CEI) film. To verify CEI film, we first evaluate the capacity retention at 55°C for 100 cycles. As presented in Fig. 3d, NCTa1–CeO_2_, NCTa1 and NC retain 91.8%, 87.5% and 71.4% of their initial capacities, respectively. The galvanostatic intermittent titration technique (GITT) curves (Supplementary Fig. S19) and calculated Li^+^ diffusion coefficients (Fig. 3e) display that NCTa1 and NCTa-CeO_2_ have better Li-ion migration kinetics than NC. NCTa-CeO_2_ shows almost the same lithium diffusion kinetics as NCTa1 because of its thin CEI film and decreased side reactions. Li-ion diffusion coefficients drop rapidly at 4.15 V due to lattice distortion caused by H2–H3 phase transition [31], which is obviously alleviated for NCTa1 and NCTa1–CeO_2_. This phenomenon indicates that modified Ni-rich cathodes can mitigate the H2–H3 phase transition. The electrochemical impedance Nyquist plots at different cycles are provided in Supplementary Fig. S20, and the corresponding equivalent circuit is displayed in Supplementary Fig. S21. The corresponding fitting data are shown in Fig. 3f and Supplementary Table S4. NCTa1–CeO_2_ shows the smallest surface-film impedance (*R_sf_*) of 12.4 Ω at the 10th cycle in comparison with NCTa1 (26.6 Ω) and NC (73.4 Ω). The value has no obvious increase for NCTa1–CeO_2_ in the 100th cycle, but 48.9 Ω for NCTa1 and 123.7 Ω for NC. These data indicate that the CeO_2_ coating is helpful in forming thinner and more stable CEI films with reduced parasitic reactions. Meanwhile, the initial charge-transfer impedance (*R_ct_*) values of NCTa1–CeO_2_ and NCTa1 are almost the same, much lower than those of NC. After 100 cycles, NCTa1–CeO_2_, NCTa1 and NC give increased amplitudes of 56.3, 133.4 and 365.2 Ω, respectively. These data imply that NCTa1–CeO_2_ possesses the highest structural integrity. The well-maintained voltage plateau and reversible phase transition during cycling (Supplementary Fig. S22) further verify the superior electrochemical performance and cycling stability of NCTa1–CeO_2_.

To further evaluate its electrochemical performance in practical applications, a pouch-type full cell is assembled using NCTa1–CeO_2_ cathodes and commercial graphite anodes. As shown in Fig. 3g, in a wide voltage range of 2.7–4.5 V, the capacity retention of this pouch cell can reach 90.9% after 1000 cycles at 1C with only 0.091% attenuation per cycle. The coulombic efficiency remains above 99.9% during the whole cycles. The charge–discharge curves also illustrate the well-maintained voltage plateau during the long-term operation (Supplementary Fig. S23). A comprehensive comparison of the electrochemical performance between NCTa1–CeO_2_ and the reported Ni-rich cathodes is illustrated in Fig. 3h and Supplementary Table S5. Impressively, the NCTa1–CeO_2_ displays almost the highest specific capacity whilst maintaining superior cycle stability.

**Figure 4.**
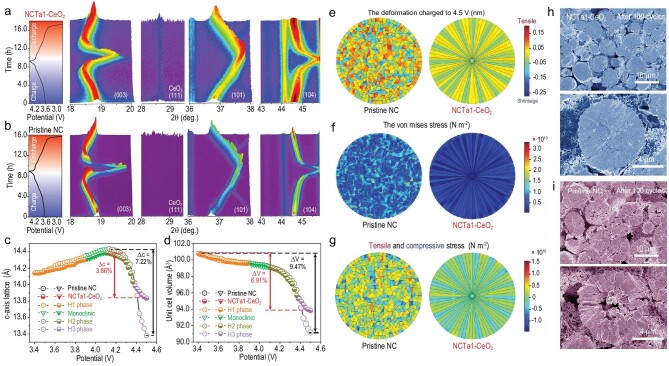
Crystal stability and stress distribution during delithiation or lithiation. (a and b) 3D *in situ* XRD contour plots of (003), (101) and (104) peaks; (c and d) corresponding variations of *c*-axis lattice parameters and unit cell volumes; (e–g) distributions of volume deformation, von Mises stress, and tensile and compressive stress when charging to 4.5 V; and (h and i) cross-sectional SEM images after 100 cycles for NCTa1–CeO_2_ and pristine NC.

To investigate the dynamic crystal structure evolution during delithiation or lithiation, electrochemical *in situ* XRD measurements of pristine NC and NCTa-CeO_2_ are conducted at 0.2C within 2.7–4.5 V. The contour plots of the partial regions and the corresponding discharge–charge curves are shown in Fig. 4a and b. The (003) peak in both cathodes first moves in the low-angle region after delithiation, corresponding to the phase transition from the hexagonal phase (H1) to the monoclinic phase (M). When the potential reaches to 4.05 V, the peak gradually returns to the high-angle region; i.e. the monoclinic phase turns into another hexagonal phase (H2). The new hexagonal phase (H3) appears once the potential exceeds 4.19 V with the rapid shift of the Bragg diffraction peak to a high angle [32]. During H2–H3 phase transition, the (003) peak shows a smooth and small shift for NCTa1–CeO_2_ whereas there is a sudden and huge move for pristine NC. The (101) and (104) peaks exhibit a similar phenomenon. Remarkably, the CeO_2_ coating layer has good chemical stability because the characteristic peak remains unchanged during delithiation or lithiation. According to the *in situ* XRD data, the lattice parameter fluctuation versus the charging potential in the *c*-axis direction is illustrated in Fig. 4c. The lattice of the two samples slightly expands before 4.05 V because the increased electrostatic repulsion between oxygen layers broadens the spacing of Li layers after delithiation. In the subsequent 4.05–4.19 V, further Ni^4+^ generation reduces the Ni–O bond length with a small lattice contraction. When charged above 4.20 V, dramatic lattice shrinkage occurs because the Ni: 3d–O: 2p orbital hybridization at such high potentials results in an abundant charge loss of oxygen anions with further Ni^4+^ accumulation. Electron-deficient oxygen ions notably decrease the electrostatic repulsion between the oxygen layers, leading to a sharp shrinkage of the Li layer and the *c*-axis lattice [14]. The maximum shrinkage ratio (Δ*c*) for pristine NC reaches 7.22%, whereas that for NCTa1–CeO_2_ is only 3.86%. The change in the unit cell volume of NCTa1–CeO_2_ also decreases from 9.47% to 6.91%, as illustrated in Fig. 4d. The *a*-axis lattice parameters of the two cathodes continuously decrease with a similar amount of shrinkage (Supplementary Fig. S24): 2.26% for pristine NC and 2.18% for NCTa1–CeO_2_. These results indicate that Ta doping significantly mitigates the charge depletion of lattice oxygen, thereby enhancing electrostatic repulsion interaction and alleviating the dramatic contraction of the *c*-axis lattice.

The aforementioned lattice parameter and unit cell volume shrinkages inevitably lead to the anisotropic volume change of primary particles with the generation of internal mechanical stresses, thus aggravating the cracking of the particle. To deeply assess mechanical stability, finite element analysis (FEA) is conducted to simulate the strain and stress inside pristine NC and NCTa1–CeO_2_ at various charge states. The particle models are built according to the SEM and TEM observations with a diameter of 10 μm for FEA, as shown in Supplementary Fig. S25. When charged to 4.0 V, the volume deformations of both cathodes are nearly identical because of their similar lattice parameter change (Supplementary Fig. S26). However, NCTa1–CeO_2_ shows the average von Mises equivalent stress of 3050 MPa and the standard deviation of 1238 MPa, which are smaller than the von Mises equivalent stress of 3160 MPa and the standard deviation of 1560 MPa of pristine NC. These data demonstrate that radially oriented primary particles can effectively dissipate internal mechanical stress and avoid local stress concentration. When the cathode is further charged to 4.5 V, the greatly decreased lattice and unit cell volume shrinkages give NCTa1–CeO_2_ a smaller volume deformation in all regions of secondary particles than NC, as shown in Fig. 4e. The comparison of von Mises equivalent stress between the cathodes is displayed in Fig. 4f. The average value and standard deviation of the equivalent stress are only 2080 and 1626 MPa for NCTa1–CeO_2_ and 8220 and 3929 MPa for pristine NC, respectively. The alleviation of mechanical internal stress in NCTa1–CeO_2_ is largely due to the synergistic effect of their small crystal deformation and orientation of primary particles. To further unveil the real stress distribution within particles, the aforementioned equivalent stress is further divided into tensile (positive value) and compressive stress (negative value), as illustrated in Fig. 4g. In contrast to pristine NC, NCTa1–CeO_2_ exhibits significantly reduced standard deviations of tensile and compressive stresses by 72.0% (from 2219 to 622 MPa) and 63.5% (from 2444 to 900 MPa), respectively, verifying the effective mitigation of stress concentration of secondary particles. The corresponding cross-section SEM images after 100 cycles are displayed in Fig. 4h and i. The NCTa1–CeO_2_ exhibits negligible structural damage without obvious cracking. On the contrary, the cracking of pristine NC can be observed from the centre of a particle to its surface. These cracks facilitate electrolyte infiltration. The newly formed electrolyte–electrode interface worsens the electrochemical performance.

**Figure 5.**
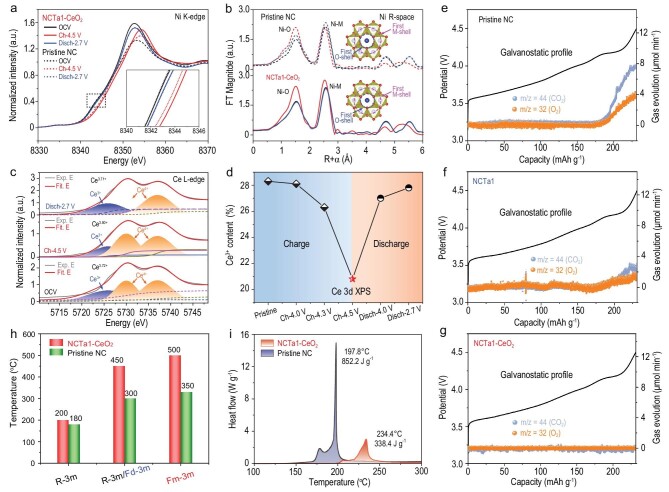
Retention mechanism of lattice oxygen and enhancement of thermal stability. (a) *Ex situ* Ni *K*-edge X-ray absorption near edge structure (XANES) spectra; (b) *K*^2^-weighted FT magnitudes of Ni *K*-edge extended X-ray absorption fine structure (FT-EXAFS) for pristine NC and NCTa1–CeO_2_; (c) *ex situ* Ce *L*-edge XANES spectra; (d) Ce^3+^ content at various potentials of NCTa1–CeO_2_; (e–g) *operando* differential electrochemical mass spectrometry (DEMS) data of pristine NC, NCTa1 and NCTa1–CeO_2_; (h) *in situ* high-temperature XRD patterns; and (i) differential scanning calorimeter (DSC) profiles of pristine NC and NCTa1–CeO_2_ after charging to 4.5 V.

The *ex situ* X-ray absorption spectroscopy (XAS) spectra are conducted at Ni *K*-edge and Ce *L*-edge to investigate the charge compensation mechanism and local structure evolution during delithiation or lithiation. Figure 5a displays the normalized X-ray absorption near the edge structure spectra of Ni *K*-edge for pristine NC and NCTa1–CeO_2_ at different potentials. Compared with that at the open circuit voltage (OCV) state, the white line of Ni shifts to high energy at the fully charged state and then back to low energy after discharging, which corresponds to the oxidation and reduction of Ni ions [33]. As illustrated in the inset of Fig. 5a, the white line of Ni for NCTa1–CeO_2_ exhibits higher energy than that of pristine NC when the cathode is charged to 4.5 V, implying a higher valence state of Ni ions after delithiation. This implication is in good agreement with the aforementioned analysis. The corresponding FT extended X-ray absorption fine structure spectra of the two samples are presented in Fig. 5b. The first main peak at ∼1.51 Å is assigned to the Ni–O shell, whereas the second peak at ∼2.52 Å represents a six-coordinated Ni–M shell contribution [34]. The corresponding structures are illustrated in the inset of Fig. 5b. The two peaks move to a low angle with enhanced intensity and then go back to the beginning at the end of a full cycle during delithiation or lithiation. The peaks at states of OCV and complete discharge for the NCTa1–CeO_2_ exhibit better overlapping than those of pristine NC at OCV and a discharge of 2.7 V, signifying a higher electrochemical reversibility. The wavelet transformed extended X-ray absorption fine structure also verifies this fact (Supplementary Fig. S27) in which NCTa1–CeO_2_ shows shorter Ni–O bond length and higher Ni ion valence state than pristine NC when charged to 4.5 V. The curve fittings for the *R*-space of the Ni *K*-edge extended X-ray absorption fine structure (EXAFS) are used to quantify the local environment differences of the two samples during delithiation or lithiation, as displayed in Supplementary Fig. S28 and Supplementary Table S6. In the OCV state, the average Ni–O coordination number (CN) of both cathodes is ∼5.3. The CN is lower than the standard octahedron of NiO_6_ mainly due to the Li loss during annealing and the difficulty in fully oxidizing Ni to the trivalent state, which is in agreement with the reports in the literature [35,36]. When charged to 4.5 V, the CN of NCTa1–CeO_2_ decreases to 5.1, which is higher than that of pristine NC (4.9), indicating the effective suppression of lattice oxygen escape. Impressively, the Ni–O CN of NCTa1–CeO_2_ can return to 5.3 when discharged to 2.7 V, whereas no change is observed for pristine NC.

To further investigate the role of the CeO_2_ layer on oxygen loss, the XAS spectra at the Ce *L*-edge under different potentials with the Gaussian and arctangent function fitting are shown in Supplementary Table S7 and Supplementary Fig. S5c [37,38]. The average valence state of Ce ions increases from +3.72 to +3.80 from OCV to the fully charged state because CeO_2_ captures a small amount of oxygen atoms that escape from the lattice. When discharged to 2.7 V, the valence state of Ce ions goes back to +3.71. The Ce 4f XPS spectra at different potentials (Supplementary Fig. S29) are also applied to demonstrate the valence state change, and the results are provided in Fig. 5d. The same observation is obtained with the Ni–O CN. These results indicate that the CeO_2_ layer can capture a small amount of oxygen whilst charging and feeding the oxygen back during the subsequent discharge process. The reversible lattice oxygen during delithiation or lithiation blocks the aggravation of parasitic reactions (Supplementary Fig. S30) and alleviates the degradation of the crystal structure (Supplementary Fig. S31). The *operando* differential electrochemical mass spectrometry is further applied to clarify the gas production of pristine NC, NCTa1 and NCTa1–CeO_2_ during delithiation (Fig. 5e–g). The O_2_ signal generally appears at the beginning of the H2–H3 phase transition (4.19 V). Meanwhile, the CO_2_ signal can be detected from the parasitic reactions of O_2_ and electrolytes. Abundant O_2_ and CO_2_ are generated in pristine NC, which are significantly reduced in NCTa1. Notably, almost no gas generation is observed in NCTa1–CeO_2_. These observations are in good agreement with the aforementioned analysis.

Thermal stability is also evaluated. Supplementary Fig. S32 shows the *in situ* high-temperature XRD curves of pristine NC and NCTa1–CeO_2_ when charged to 4.5 V. The data are illustrated in Fig. 5h. The layered structure is well maintained even under 100% SOC. As the temperature rises, the Ni ions in the TM layer gradually move into the vacancy of the lithium layer, which causes the generation of spinel and rock-salt phases. NCTa1–CeO_2_ appears during the spinel phase at 200°C, but only at 180°C for pristine NC. This finding is mainly reflected in the merger of the split (108) and (110) peaks into one (440) peak [39]. The spinel structure is fully converted into the rock-salt phase at 500°C for NCTa1–CeO_2_, but only at 350°C for pristine NC. Differential scanning calorimetry is performed to intuitively compare the thermal stability, as shown in Fig. 5i. NCTa1–CeO_2_ displays an exothermic reaction peak at 234.4°C, which is 36.6°C higher than pristine NC. Furthermore, the total heat release of NCTa1–CeO_2_ is only 338.4 J g^−1^, which is much lower than the 852.2 J g^−1^ of pristine NC. These results indicate that NCTa1–CeO_2_ possesses superior thermal stability than that of pristine NC. In general, the adverse phase transformation and interfacial parasitic reactions easily cause much heat generation and even thermal runaway. Restraining lattice oxygen escape can alleviate the adverse phase transformation from the layer structure to the spinel or rock-salt phase. Such restriction avoids electrolyte decomposition and corrosion to active materials due to limited oxygen radicals.

## CONCLUSIONS

In summary, we demonstrate the one-step synthesis of Ta-doped and CeO_2_-coated Ni-rich cathodes to fully restrain the lattice oxygen escape when operated at high voltage. Ta is doped into octahedral interstices in TM layers and simultaneously induces the growth orientation of primary particles. As an electron donor, Ta doping alleviates the charge depletion of lattice oxygen and stabilizes it due to high Ta–O bonding energy. The remaining oxygen escape is further captured by the CeO_2_ coating layer and then fed back to the lattice of the cathode during the discharge process. The synergy significantly reduces the parasitic reactions with high coulombic efficiency. The electron-enriched lattice oxygen also effectively avoids the sudden contraction of lattice parameters with strong electrostatic repulsion between oxygen layers, hence reducing the generation of strain and stress in secondary particles. Radially oriented primary particles are helpful in dissipating mechanical internal stress to prevent stress concentration and cracking. As a consequence, the dual-modified cathode exhibits a capacity of 231.3 mAh g^−1^ with a high ICE of 93.5% and 136.0 mAh g^−1^ even at 20C. A superior cyclic performance is also achieved where the capacity retention is 96.8% at 25°C and 91.8% at 55°C after 100 cycles. In a pouch-type full cell, 90.9% of its initial capacity is still maintained at 1C after 1000 cycles at 2.7–4.5 V. The present dual-track strategy can be popularized in other cathode materials that exhibit the detriment of unstable lattice oxygen.

## METHODS

### Materials preparation

Spherical Ni_0.9_Co_0.1_(OH)_2_ precursors were prepared using a co-precipitation method, which has been described in our previous studies [32]. The obtained Ni_0.9_Co_0.1_(OH)_2_ precursor was mixed with the stoichiometric LiOH·H_2_O (molar ratio of lithium to TM = 1.02). The mixture was pre-calcined at 500°C for 5 h, followed by sintering at 720°C for 15 h in a pure oxygen atmosphere to obtain the pristine Ni-rich oxides (pristine NC). To synthesize Ta-doped and CeO_2_-coated Ni-rich cathodes (NCTa1–CeO_2_), 1.0 g of Ni_0.9_Co_0.1_(OH)_2_ precursors was dispersed in 50 mL of absolute ethanol; then, stoichiometric Ta ethoxide and Ce isopropoxide were simultaneously added to the aforementioned suspension liquid. After 30 min of ultrasound, the suspension liquid was stirred at 60°C until the solvent was completely evaporated to obtain Ta and Ce compound-coated Ni_0.9_Co_0.1_(OH)_2_ precursors. Next, the modified precursor was lithiated and calcinated in the same condition as pristine NC to prepare NCTa1–CeO_2_. To obtain Ta-doped Ni-rich cathodes, only tantalum ethoxide was added to the suspension liquid containing the Ni_0.9_Co_0.1_(OH)_2_ precursors. Other conditions were consistent with the preparation of NCTa1–CeO_2_. NCTa0.5, NCTa1, NCTa1.5 and NCTa2 were defined when the Ta content was 0.5, 1.0, 1.5 and 2 mol%, respectively.

## Supplementary Material

nwac166_Supplemental_File
