# Supplementary material for: Restraining the escape of lattice oxygen enables superior cyclic performance towards high-voltage Ni-rich cathodes
Source: Natl Sci Rev. 2022 Aug 18;10(1):nwac166. doi: 10.1093/nsr/nwac166 (PMC9843122; doi:10.1093/nsr/nwac166)
Supplement: nwac166_Supplemental_File [file nwac166_supplemental_file.pdf]

Supplementary information

Restraining lattice oxygen escape enables superior cyclic performance toward high-voltage Ni-rich cathodes

Haifeng Yu[1], Huawei Zhu[1], Hongliang Jiang[2], Xiaozhi Su[3], Yanjie Hu[1], Hao Jiang[1,2,]* and Chunzhong Li[1,2,]*

[1]Key Laboratory for Ultrafine Materials of Ministry of Education, School of Materials Science and Engineering, East China University of Science and Technology, Shanghai, 200237, China

[2]Shanghai Engineering Research Center of Hierarchical Nanomaterials, School of Chemical Engineering, East China University of Science and Technology, Shanghai, 200237, China

[3]Shanghai Synchrotron Radiation Facility, Shanghai Advanced Research Institute, Chinese Academy of Sciences, Shanghai 201210, China

*__Corresponding authors__. E-mails: jianghao@ecust.edu.cn; czli@ecust.edu.cn

1 Supplementary Notes

1.1 Materials characterization

The chemical compositions of the samples were obtained by the inductively coupled plasma atomic emission spectrometer (ICP-AES, Agilent 725). The X-ray diffraction (XRD, Bruker D8 Advance) was performed to analyze the crystal structure, and the atomic occupation situations and lattice parameters were fitted by Rietveld refinement. The cathodes charged to 4.5 V were investigated by *in situ* high-temperature XRD (HT-XRD, from 30℃ to 600℃) to assess their thermal stability of crystal structure. To monitor phase transition during the electrochemical process, an electrochemical *in situ* XRD test (*in situ* XRD, Rigaku Ultima IV) was carried out by connecting with a LANDCT2001A test system. Concretely, the modified coin-type half-cells that can pass through X-rays were performed galvanostatic charging-discharging procedure at a current of 40 mA g^{-1}, and the system will automatically record the diffraction patterns around every 3.5 min. The field emission scanning electron

microscopy (FESEM; GeminiSEM 500) was used to observe the morphologies of the materials, and cross section of electrode after cycling was fabricated by triple ion beam milling system (Leica EM TIC 3X). The microstructure and element distribution were investigated by high resolution transmission electron microscopy (HRTEM; FEI Talos F200X) and double aberration-corrected scanning transmission electron microscopy (Cs-STEM; FEI Themis Z) with accessory of energy disperse X-ray spectroscopy (EDS), and the samples were prepared by focused ion beam etching technique (FIB, TESCAN GALA 3). The surface chemical valence states of the relevant elements were analyzed by X-ray photoelectron spectroscopy (XPS; ESCA PHI500C). In-situ differential electrochemical mass spectrometry (DEMS) were carried out at DEMS-QAS 100 (Linglu Instruments (Shanghai) Co. Ltd.), and the corresponding cell was Swagelok configuration assembled in the glove box. The thermal stability was tested via differential scanning calorimeter (DSC; NETZSCH DSC204) using delithiatied cathode with existence of electrolyte. XAFS measurements at Ni *K*-edge (transmission mode) and Ce *L*-edge (fluorescence mode) were performed at BL14W1 in Shanghai Synchrotron Radiation Facility (SSRF, shanghai, China). The raw data analysis was performed using IFEFFIT software package according to the standard data analysis process. The spectra were calibrated, averaged, pre-edge background subtracted, and post-edge normalized using Athena program in IFEFFIT software package. The Fourier transformation of the k^3-weighted EXAFS oscillations, $k^3 \cdot \chi(k)$, from k space to R space were performed over range of 2.5-14.2 Å^{-1} to obtain radial distribution function. EXAFS data were fitted by Artemis program in IFEFFIT.

1.2 Electrochemical test

The cathode materials were mixed with polyvinylidene difluoride (PVDF) and Super-P conductive carbon having a mass ratio of 8:1:1 in a suitable amount of N-methyl-2-pyrrolidone (NMP) to make slurry. Single side coated electrodes were prepared

by casting the slurry onto aluminum foil. After drying in the vacuum oven at 120℃ for 12 h, the coin cell electrodes were punched with a 1.0 cm diameter punch. Standard coin-type 2016 cells were assembled in a argon-filled glovebox with lithium metal as the negative electrode and polypropylene membrane (Celgard-2400) as the separator, and the electrolyte was 1.2 M $LiPF_6$ in EC : EMC (3 : 7 v/v) with 2wt% VC. For the pouch cell, the loading mass of cathode was increased to 15 mg cm^{-2}, and loading mass of commercial graphite anode was also controlled to ensure the suitable N/P ratio of 1.1. After rolling and slitting (6.1*4.3 cm^2 for each piece), the cathode and anode were stacked in sequence and separated by the separator to obtain dry cell. Next, the dry cell was packaging using aluminum-plastic film and then the electrolyte was injected into it to obtain a pouch-cell. Amounts of electrolyte used in coin cell and in pouch cell are 100 μL and 8 g, respectively. The galvanostatic charge and discharge tests within a voltage range of 2.7-4.5 V of coin- and pouch-cells were performed with LANDCT2001A Neware test systems, respectively. The value of 1 C was 200 mA g^{-1}. Galvanostatic intermittent titration technique (GITT) test was carried out in LANDCT2001A test system with a 2 h relaxation time of 2 h after charging for 15 min at 0.2 C. Cyclic voltammetry (CV) curves and electrochemical impedance spectra (EIS) were performed in an Autolab PGSTAT302N electrochemical workstation. To research the cathodes after electrochemical reaction, the coin batteries were disassembled in an argon filled glovebox, and absolutely dried in a heating plate of 60℃ after washing with pure dimethyl carbonate.

1.3 Theoretical Calculations

In this work, the first-principles calculation steps are completed through geometry optimization, self-consistent field (SCF) and charge calculation. All calculations are performed by VASP-6.1.0[1], combined with the PBE functional[2, 3] under the generalized gradient approximation (GGA)[4] in the frame of density functional theory (DFT) with D3

dispersion correction[5], combined with the projector augmented wave (PAW)[6], and the plane wave basic sets cut off energy is 500 eV. Among them, K point adopts the density of 9*9*1. To assess the doping and surface energies of pristine and doped $LiNiO_2$, the 3×3×1 supercell with appropriate lattice parameters was utilized, and the tantalum ions were substituted nickel sites in transition layers with atomic ratio of 1%. Besides, the delithiated $Li_{0.1}NiO_2$ and $Li_xNi_{0.99}Ta_{0.01}O_2$ systems was modeled by randomly extracting 90% lithium from above $LiNiO_2$ and $LiNi_{0.99}Ta_{0.01}O_2$ configurations. The doping formation energy (ΔE) of tantalum or cerium ion was defined as $\Delta E=E_{doped\ system}+\mu_{substituted\ atom}-E_{pristine\ system}-\mu_{dope\ atom}$, where $E_{doped\ system}$ and $E_{pristine\ system}$ were the energy of doped and pristine $LiNiO_2$, respectively. $\mu_{substituted\ atom}$ and $\mu_{dope\ atom}$ were the chemical potentials of substituted and replaced elements, respectively. The surface energy (γ) of each surface was defined as the energy difference between the slab and bulk structures. It was calculated via the following equation:

$$\gamma_{pristine} = \frac{1}{2A}\left[E_{Surface}^{Pristine} - E_{bulk}^{pristine} + (N_{Ni} - N_{Li})\mu_{Li}\right] \text{ and}$$

$$\gamma_{Ta-doped} = \gamma_{doped} - \gamma_{pristine}$$
$$= \frac{1}{A}\left[E_{Surface}^{Ta-doped} - E_{bulk}^{Ta-doped} + (N_{Ni} - N_{Li})\mu_{Li}\right] - \frac{1}{2A}\left[E_{Surface}^{Pristine} - E_{bulk}^{pristine} + (N_{Ni} - N_{Li})\mu_{Li}\right]$$

, where the γ_{doped} is the surface energy of the asymmetric slab with Ta-doped and pristine sides. E_{surf} and E_{bulk} are the total energy of surface and bulk structure, respectively. N_{Ni} and N_{Li} are number of nickel and lithium on the surface, and μ_{Li} is the chemical potential of lithium.

1.4 Method and model of finite element analysis

The coupled mechanical and thermal modules in finite element analysis were carried to obtain the volume deformation and internal stress of cathodes with different microstructure at

different stage of charge. The elastic modulus, Poisson's ratio and density of the material were set to 116 GPa, 0.25 and 2.75 g cm^{-3}, respectively. The initial displacement and speed were both set to 0 and the boundary conditions were fixed constraint boundary conditions. The relative tolerance of convergence condition was sat as 0.001. The grain orientation was random (in the range of 0-360 °). The transformation matrix was used to represent the relationship between the local expansion/contraction and the global expansion/contraction: $\alpha_{ij}^{L} = tr_{ki} tr_{lj} \alpha_{kl}$.

1.5 The testing process and calculation equation of GITT measurement

Before the GITT measurement, the cells were firstly galvanostatic charge/discharged for 2 cycles. GITT measurements were performed by charging/discharging the fully activated cells at a constant current (0.1 C) for an interval of 20 min followed by an open circuit stand for 2 h to allow the cell voltage to relax to its quasi-equilibrium state. The change in the steady-state voltage ΔE_s is obtained by subtracting the original voltage (E_0) from the steady-state voltage (E_s). The cell voltage increases during the current flux and the total change of cell voltage ΔE_τ can be obtained by calculating the voltage drop. Meanwhile, the process of the chemical diffusion is assumed to obey Fick's second law of diffusion. With a series of simplifications, for sufficient time interval ($\tau \ll L^2/D_{Li+}$), the equation of D_{Li+} can be written as (Equation 2):

$$D_{Li^+} = \frac{4}{\pi}\left(\frac{m_B V_m}{M_B A}\right)^2 \left(\frac{\Delta E_S}{\tau\left(\frac{dE_\tau}{d\sqrt{\tau}}\right)}\right)^2 \left(\tau << \frac{L^2}{D_{Li}{}^+}\right) \qquad (2)$$

where V_m is the molar volume of active materials, M_B and m_B are the molecular weight and mass of the host oxide, respectively, and A is the total contact area between the electrolyte and

the electrode, L is the thickness of the electrode. If sufficiently small currents and short time intervals are employed, the cell voltage is a linear function of the square root of τ, Equation 2 can be further simplified as (Equation 3):

$$D_{Li^+} = \frac{4L^2}{\pi\tau}\left(\frac{\Delta E_S}{\Delta E_\tau}\right)^2 \left(\tau << \frac{L^2}{D_{Li}{}^+}\right) \qquad (3)$$

2 Supplementary Figures

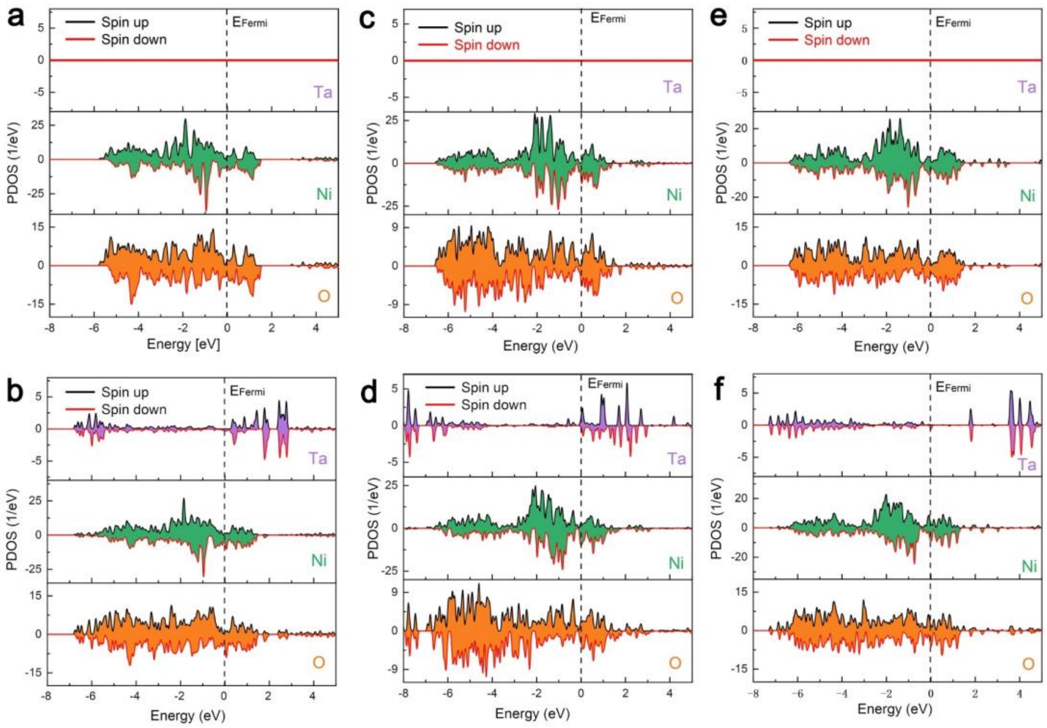

Fig. 1 The calculated projected density of states of O: 2p, Ni: 3d, and Ta: 5d for (a, b) (003), (c, d) (104) and (e, f) (012) faces of pristine $Li_{0.1}NiO_2$ and tantalum-doped $Li_{0.1}NiO_2$, and Fermi lever is set as 0 eV.

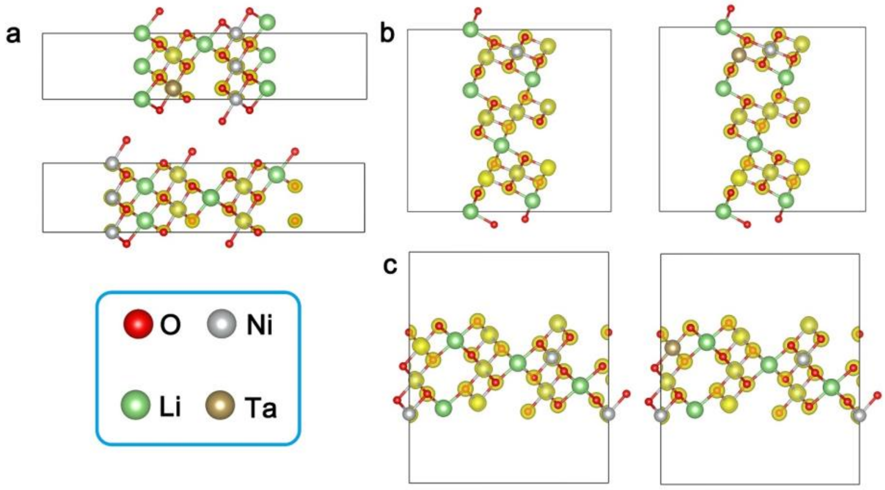

Fig. 2 Charge density distribution for (a) (003), (b) (104) and (c (012) faces of pristine $Li_{0.1}NiO_2$ and tantalum-doped $Li_{0.1}NiO_2$.

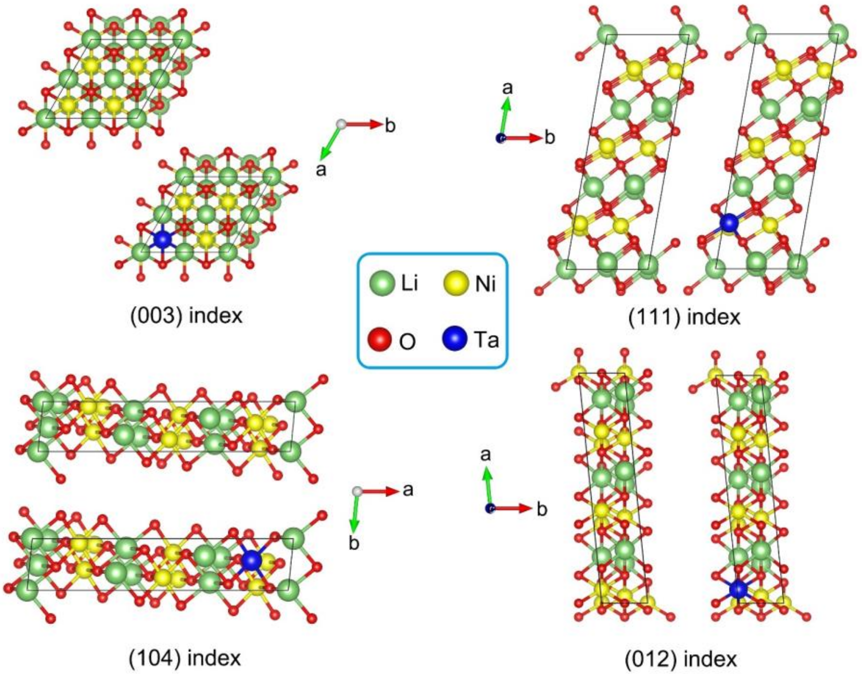

Fig. 3 The crystal structure for surface energy calculation of pristine $LiNiO_2$ and tantalum-doped $LiNiO_2$.

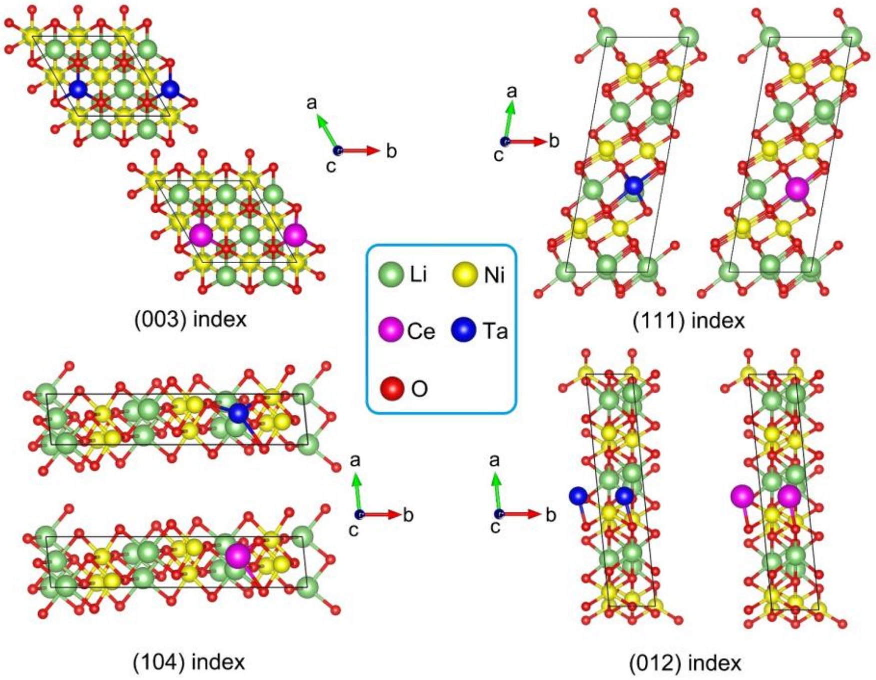

Fig. 4 The crystal structure for calculation of doping barrier energies.

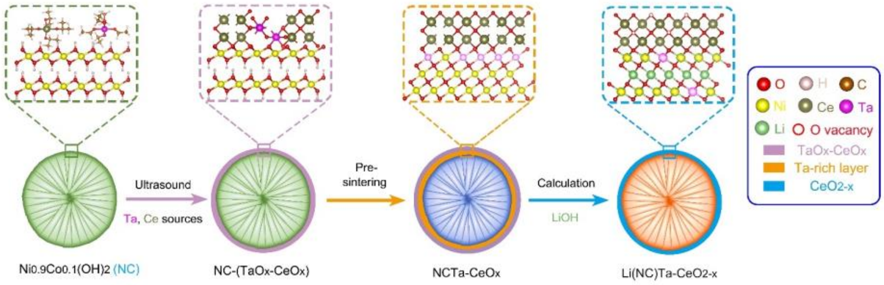

Fig. 5 Schematic illustration of the preparation prcess for simultaneously Ta-doped and CeO_2-coated Ni-rich cathode.

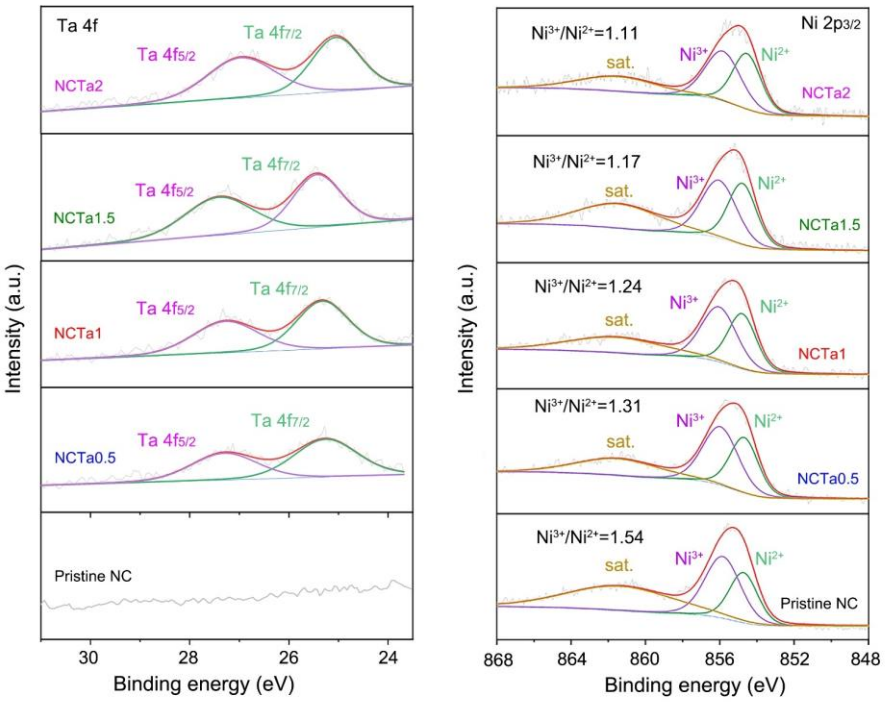

Fig. 6 Ta 4f and Ni $2p_{3/2}$ XPS spectra of the samples with different tantalum contents.

Notes: The modified Ni-rich cathodes with different Ta contents were firstly prepared to select the optimist doping content. As shown in **Supplementary Table 2**, the relative fraction of Ni, Co, and Ta are match well with our purported molar ratio according to the inductively coupled plasma atomic emission spectroscopy (ICP-AES). **Supplementary Fig. 6** displayed the X-ray photoelectron spectroscopy (XPS) results of different samples. For Ta 4f XPS spectra, the peaks at 25.2 eV and 27.1 eV are ascribed to Ta $4f_{5/2}$ and Ta $4f_{7/2}$ signal, indicating that the doped Ta ions exist in the form of Ta^{5+}[7]. Meanwhile, the intensities of the peaks gradually improve with the increase of doping content. The distinction of Ni $2p_{3/2}$ spectra for different samples was also analyzed. The specific signals of 854.6 and 855.9 eV in Ni $2p_{3/2}$ spectra confirm the coexistence of Ni^{2+} and Ni^{3+} in the samples[8]. Due to the constraint of charge balance, more Ni^{3+} is reduced to Ni^{2+} with the increase of Ta content, further corroborating the doping of high valence Ta^{5+}.

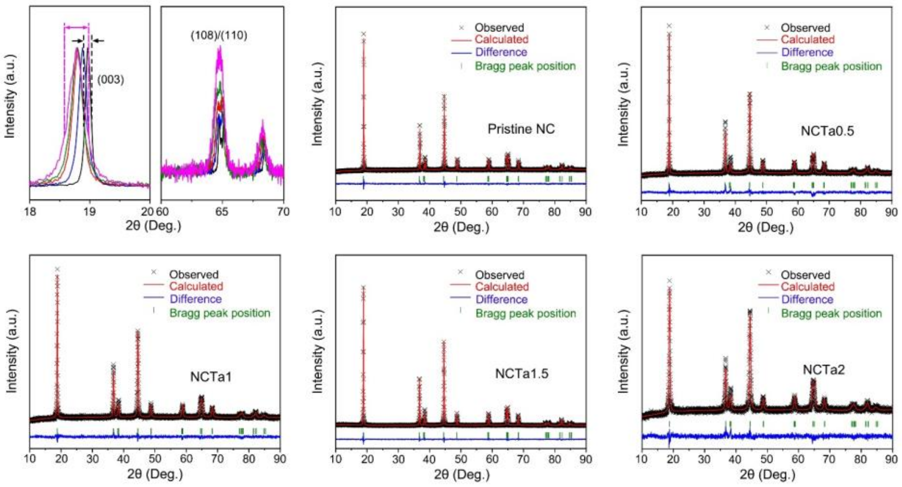

Fig. 7 Enlarged XRD patterns and XRD Rietveld refinements of the samples with different tantalum contents.

Note: The comparison of normalized (003) peaks for different samples shows that the full width at half maximum (FWHM) improve with the increasing fraction of Ta in samples, indicating the Ta doping could reduce the size of primary particles. The XRD Rietveld refinements were performed to study the detailed lattice parameters and Li/Ni disorder, and the corresponding patterns and data were shown in **Supplementary Fig. 7** and **Supplementary Table 3**. The diffraction peaks of samples are all indexed to the space group of R-3m (no. 166) and consistent well with the hexagonal $LiNiO_2$ (JCPDS 09-0063), and no impurity exists in sample. The increase of Ni^{2+} due to Ta^{5+} incorporation is beneficial to improve the lattice parameter, but the corresponding enhanced Li/Ni disorder will reduce it. Therefore, there will be an appropriate amount of doping under the influence of these opposite effects, and that is 1% (NCTa1) according to the XRD refinement results. The previous reports also evidenced that slightly improved Li/Ni disorder caused by doping of high-valence elements, like W^{6+}, Nb^{5+}, Mo^{7+}, Ta^{5+} is also advantageous for structural stability

of Ni-rich cathode.

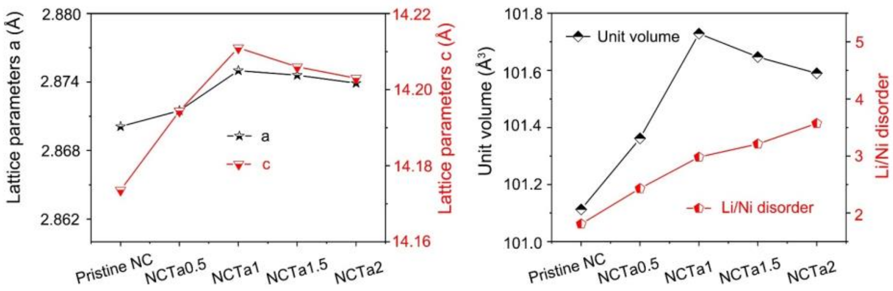

Fig. 8 The comparision of lattice parameters, unit volume and Li/Ni disorder degree according to the results XRD Rietveld refinements.

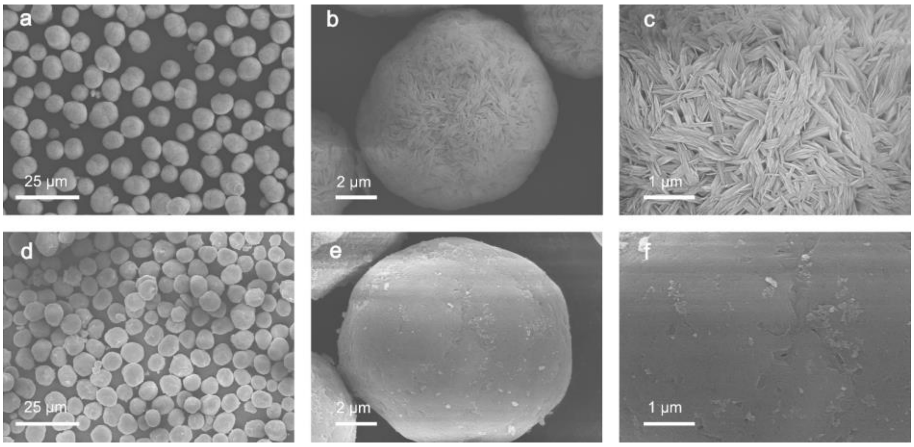

Fig. 9 SEM images of (a-c) pristine and (d-f) modified $Ni_{0.9}Co_{0.2}(OH)_2$ precursors.

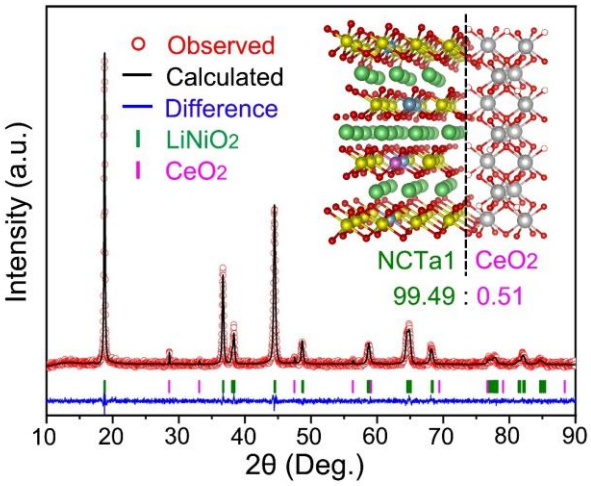

Fig. 10 XRD Rietveld refinement of NCTa1-CeO_2 sample with crystal structure diagram and weigh ratio of different components.

Note: Supplementary Fig. 10 displayed the XRD refinement pattern with corresponding crystal diagram. The main diffraction peaks are assignable to the space group of R-3m (no. 166) for hexagonal $LiNiO_2$ (JCPDS 09-0063), and the other peaks are indexed to Fm-3m space group (no. 225) for CeO_2 (JCPDS 34-0394).

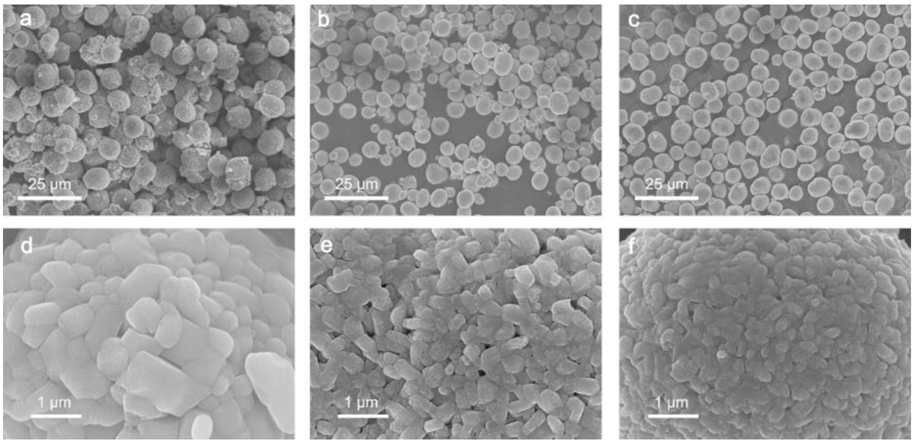

Fig. 11 SEM images of (a, d) pristine NC, (b, e) NCTa1 and (c, f) NCTa1-CeO_2.

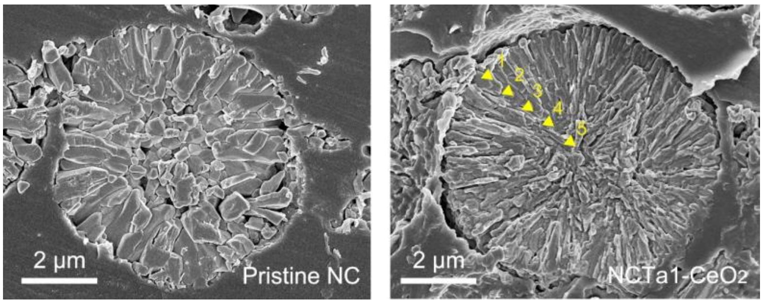

Fig. 12 Cross-sectional SEM image of NCTa1-CeO_2. The Ta content is detected at the five yellow triangles.

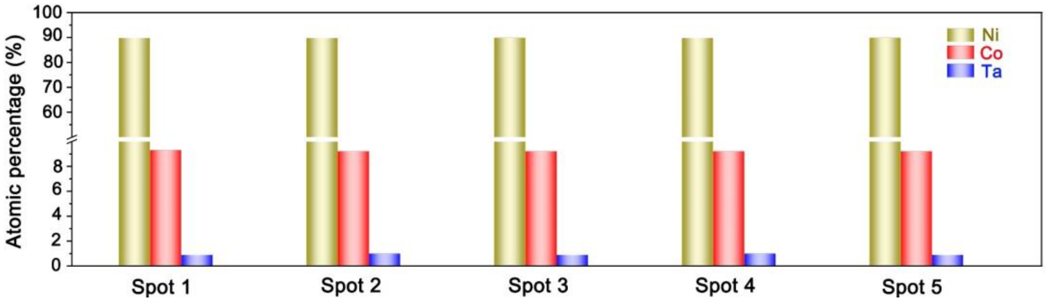

Fig. 13 The atomic percentage across the secondary particles detected by EDS point analyses.

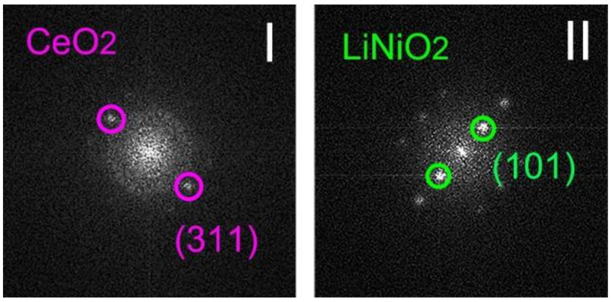

Fig. 14 Fourier transform images of surface and bulk parts in HRTEM image.

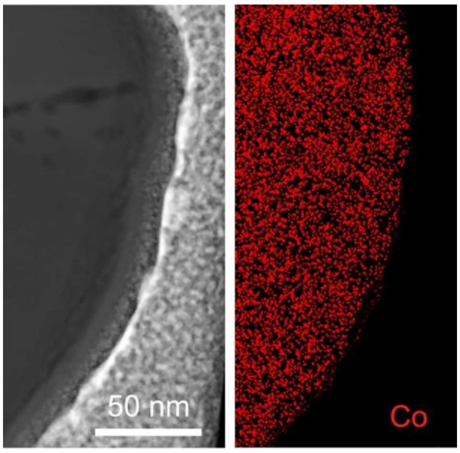

Fig. 15 HAADF image of NCTa1-CeO_2 with EDS mapping image of cobalt.

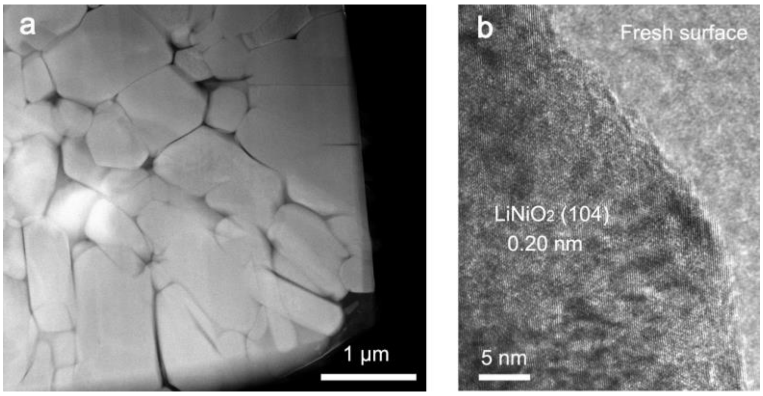

Fig. 16 STEM-HAADF and HRTEM images of pristine NC.

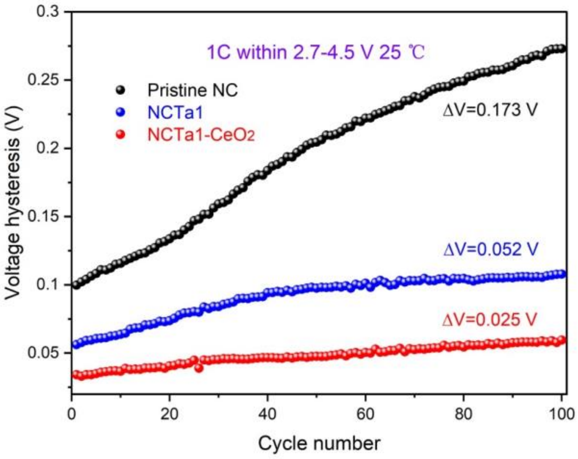

Fig. 17 The change of voltage hysteresis and energy density during cycling at 1C for pristine NC, NCTa1

and NCTa1-CeO_2.

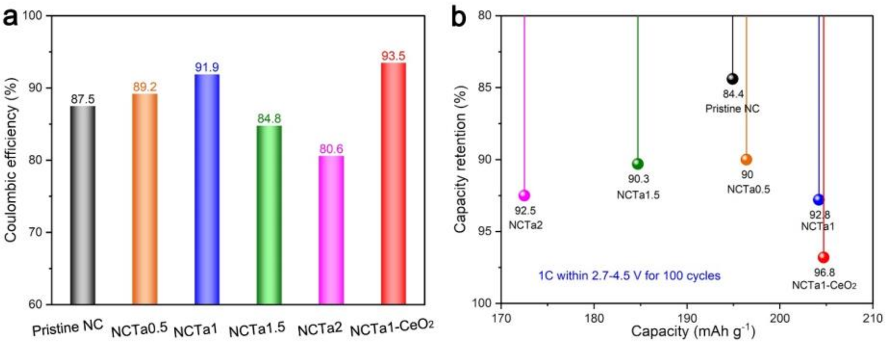

Fig. 18 The comparison of initial Columbic efficiencies and cycle retention ratio for all of samples.

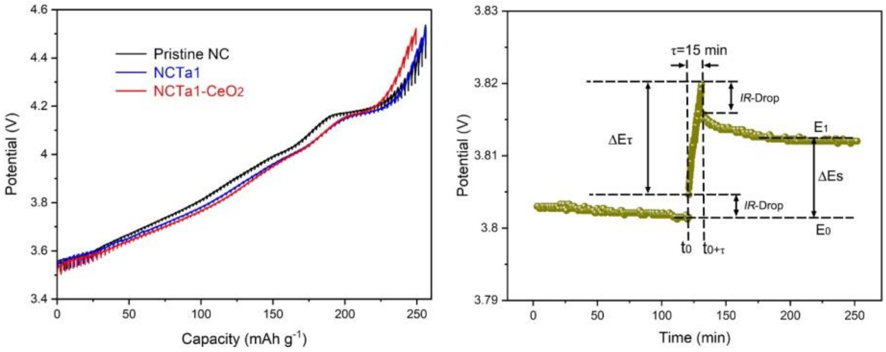

Fig. 19 GITT curves of charging curves for pristine NC, NCTa1 and NCTa1-CeO_2, and single titration at about 3.81 V.

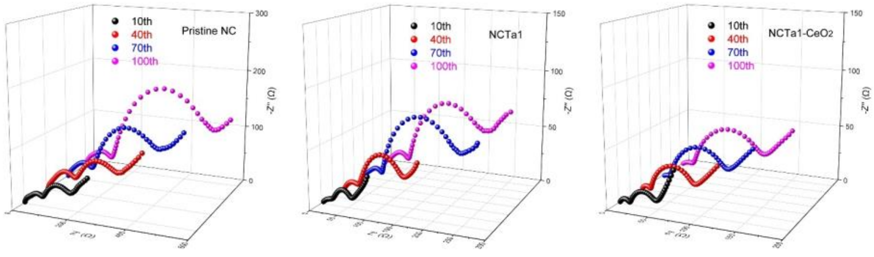

Fig. 20 Nyquist plots of electrochemical impedances at the fully charged state (4.5 V) at different cycle numbers for the pristine NC, NCTa1 and NCTa1-CeO_2.

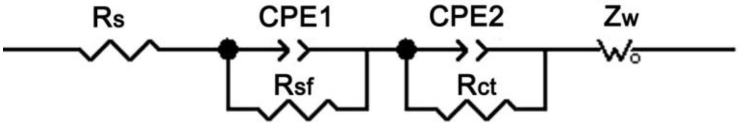

Fig. 21 The equivalent circuit for the EIS test.

Notes: According to the equivalent circuit in **Supplementary information Fig. 21**, The first semicircle in the high-medium frequency range is attributed to surface film resistances (R_{sf}), while the second semicircle at the medium frequency is identified as the charge-transfer resistance (R_{ct}) of the cathode.

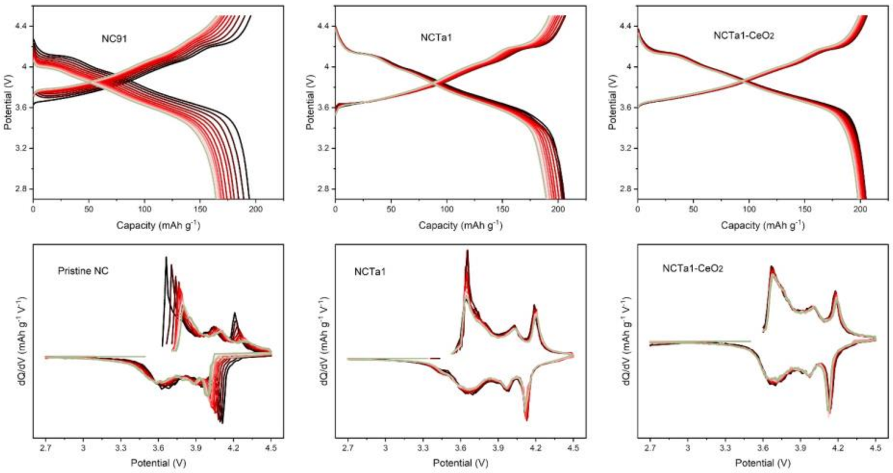

Fig. 22 The charge/discharge curves and the calculated dQ/dV curves by differentiating charge/discharge curves at different cycle numbers for pristine NC, NCTa1 and NCTa1-CeO_2.

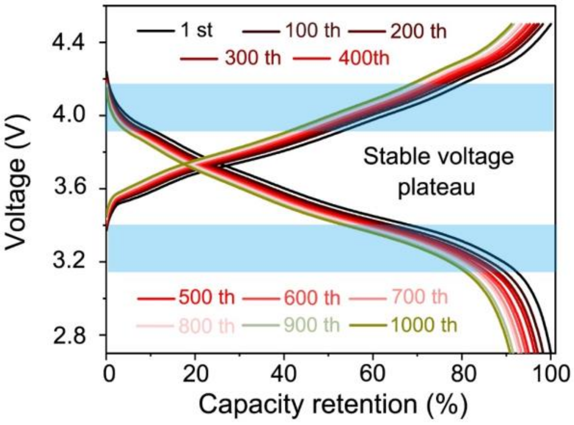

Fig. 23 The charge/discharge curves at different cycle numbers for NCTa1-CeO_2/graphite full cells.

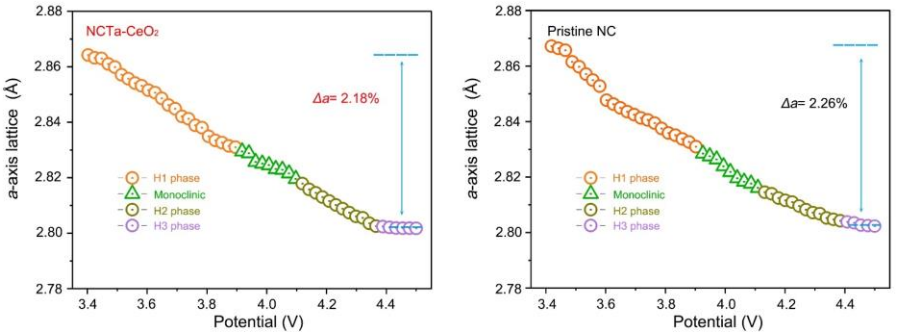

Fig. 24 Variations of *a*-axis lattice parameters as a function of charging potential.

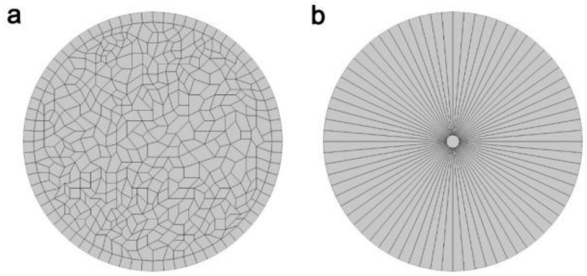

Fig. 25 2D models of (a) pristine NC and (b) NCTa1-CeO_2 for finite element analysis.

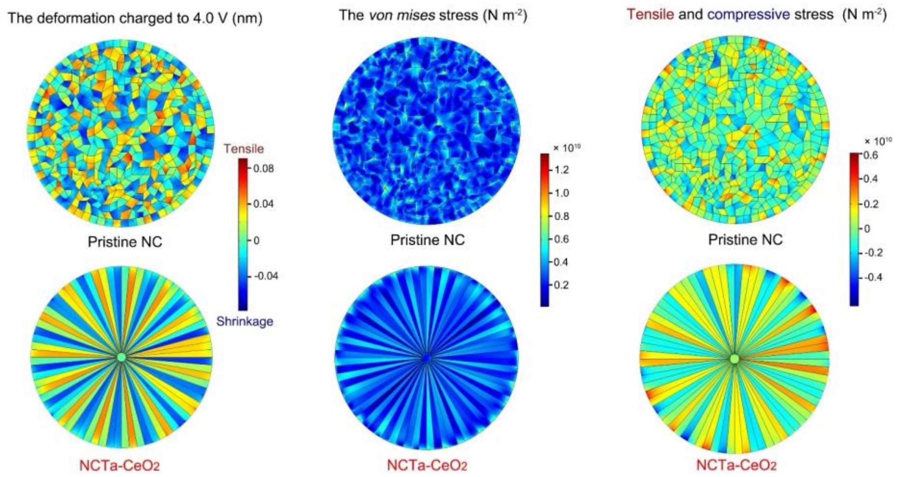

Fig. 26 The distribution of volume deformation, *Von mises* stress, tensile and compressive stress thoughout the secondary particles for pristine NC and NCTa1-CeO_2 when charging to 4.0 V.

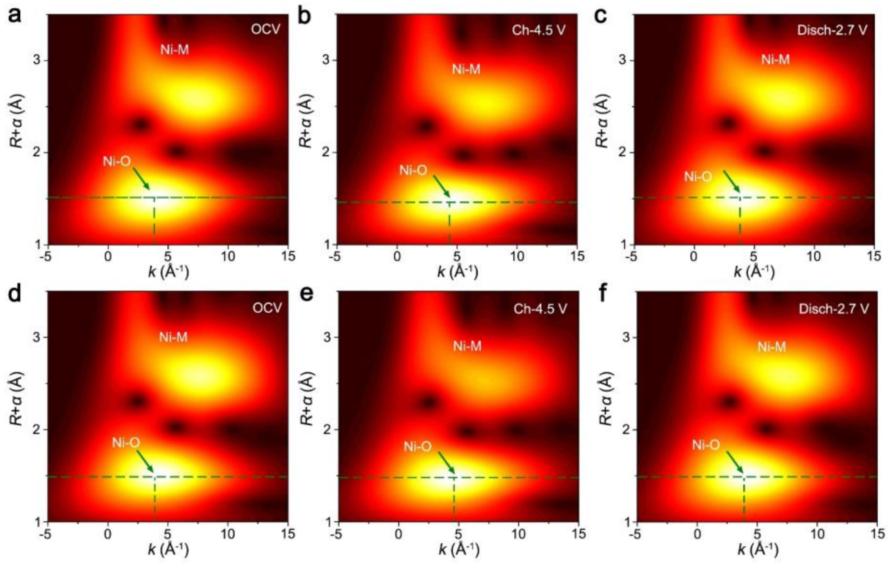

Fig. 27 Wavelet transformed extended X-ray absorption fine structure (WT-EXAFS) of Ni *K*-edge signal for (a-c) NCTa1-CeO_2 and (d-f) pristine NC.

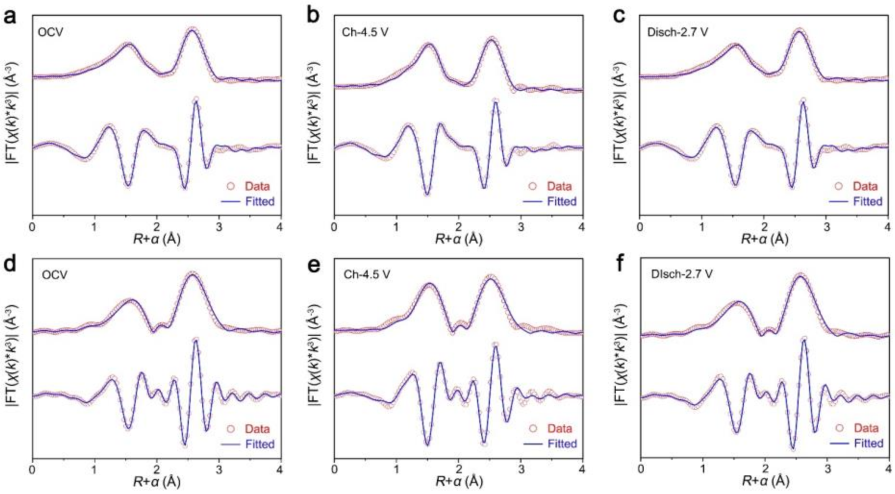

Fig. 28 Ni-O and Ni-M shell fitting of Fourier transformation of *ex-situ* Ni *K*-edge EXAFS for (a-c) NCTa1-CeO_2 and (d-f) pristine NC. Top and bottom traces are the magnitude and imaginary parts, respectively.

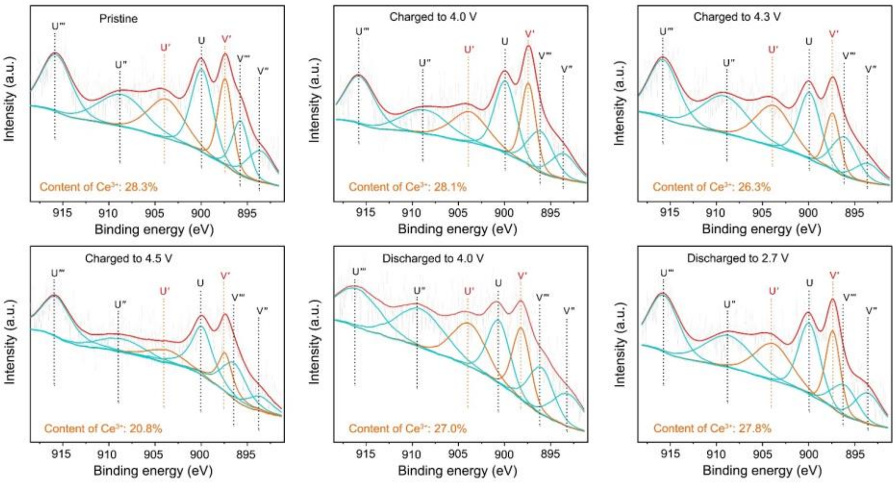

Fig. 29 Ce 4f XPS spectra of NCTa1-CeO_2 at different potential.

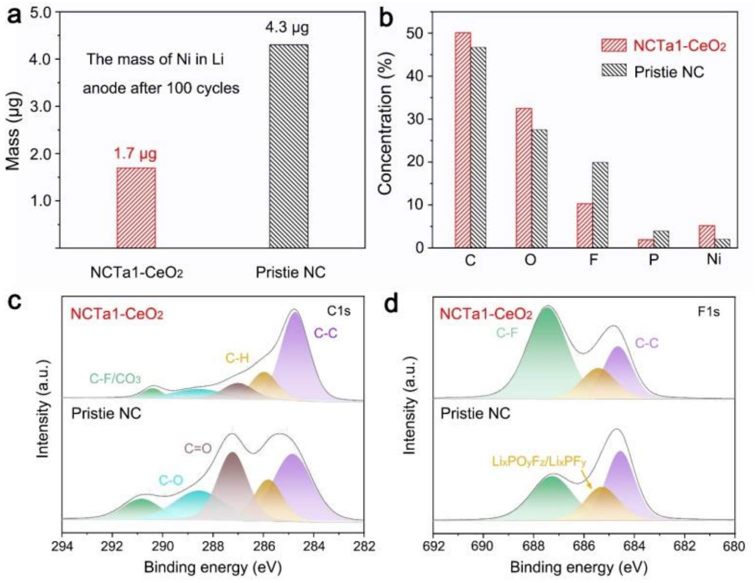

Fig. 30 (a) The amount of Ni deposited on the lithium anode, (b) chemical composition of different elements in XPS spectra, (c, d) C 1s and F 1s XPS spectra of NCTa1-CeO_2 and pristine NC after 100 cycles.

Notes: To evidence the mitigation of interfacial parasitic reaction by restraining the oxygen escape, the ICP-OES test for Li anode and XPS characterization for cathodes after cycling were carried out. As shown in **Supplementary Fig 30a**, the Ni content in the Li anode is

obviously decreased after modification (1.7 μg for NCTa1-CeO_2 and 4.3 μg for pristine NC). Besides, the elemental concentrations obtained from XPS results display the higher contents of F and P elements while the lower content of Ni before modification, suggesting more parasitic reactions and thicker CEI film on the surface of pristine NC (**Supplementary Fig. 30b**). The high-resolution XPS analysis of C 1s and F 1s peaks were shown in **Supplementary Fig. 30c, d**. The C-O, C=O bonds in C 1s peaks and the $Li_xPO_yF_z$/Li_xPF_y, LiF compounds in F 1s peaks were considered as the signal of parasitic reaction[9, 10]. Impressively, the intensities of these peaks are all reduced in NCTa1-CeO_2 compared with pristine NC.

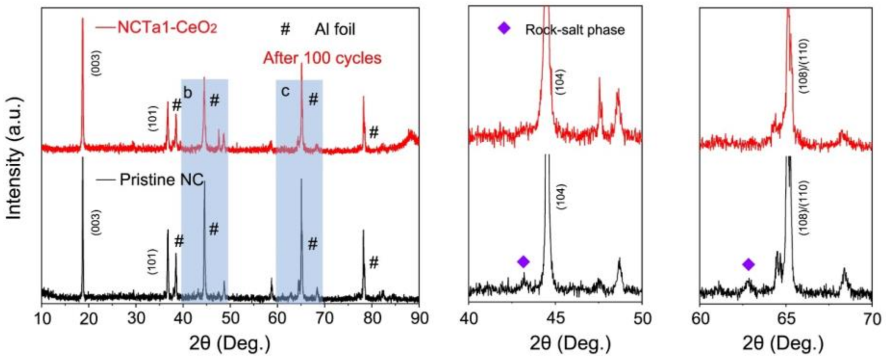

Fig. 31 XRD patterns and magnified graphs of NCTa1-CeO_2 and pristine NC after 100 cycles.

Notes: XRD patterns of NCTa1-CeO_2 and pristine NC after 100 cycles were exhibited in **Supplementary Fig. 31**. No other peak appears in the graph of NCTa1-CeO_2 except for aluminum foil and bulk layered structure. In contrast, two distinguishable peaks located at ca 43 ° and 63 ° are observed in the peaks of pristine NC, which can be indexed to the rock-salt phase with space group of Fm-3m (JCPDS: 47-1049). The structure degradation is ascribed from the escape of lattice oxygen, which will lead to the migration and reduction of nickel

ions[11].

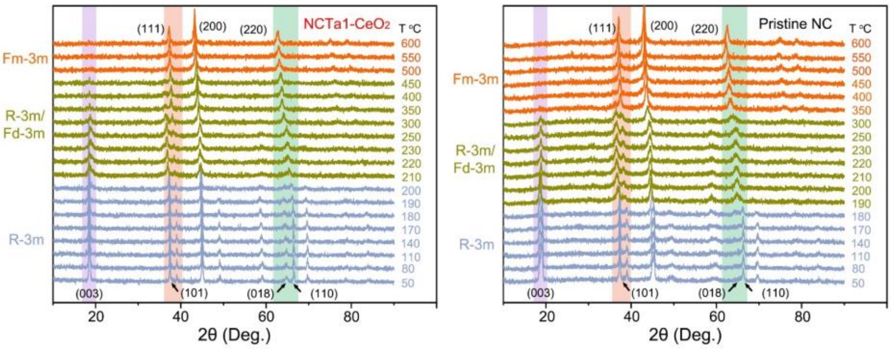

Fig. 32 Full range HT-XRD patterns of delithiated NCTa1-CeO_2 and pristine NC (charged to 4.5 V) during heating from 30 to 600 ℃.

3 Supplementary Tables

Table 1 The surface energies used for simulating the Wulff shape.

	(003)	(111)	(104)	(012)
LNO	10.63 eV	9.57 eV	13.16 eV	17.79 eV
LNO-Ta	4.65 eV	7.02 eV	12.97 eV	13.38 eV

Table 2 Chemical compositions and valence state of Ni for Pristine NC, NCTa0.5, NCTa1, NCTa1.5 and NCTa2 measured by ICP-AES and XPS.

Sample	Chemical composition obtained by ICP-AES			Ni^{3+}/Ni^{2+} obtained by XPS
	Ni	Co	Ta	
Pristine NC	0.898	0.102	-	1.54
NCTa0.5	0.895	0.099	0.005	1.31
NCTa1	0.892	0.099	0.009	1.24
NCTa1.5	0.888	0.098	0.014	1.17
NCTa2	0.884	0.098	0.018	1.11

Table 3 Rietveld refinement results of XRD data for various samples.

Sample	FWHM	$I_{(003)}/I_{(104)}$	a (Å)	c (Å)	Vol. ($Å^3$)	Li_{Ni} (%)	R_{wp} (%)	R_p (%)	χ^2
Pristine NC	0.131	1.95	2.8701	14.1736	101.113	1.81	5.24	4.15	1.232
NCTa0.5	0.168	1.87	2.8715	14.1944	101.362	2.43	6.08	4.72	1.562

NCTa1	0.200	1.82	2.8750	14.2110	101.729	2.98	5.37	4.24	1.163
NCTa1.5	0.204	1.63	2.8746	14.2060	101.647	3.21	6.46	4.59	2.006
NCTa2	0.210	1.32	2.8739	14.2030	101.590	3.57	5.89	4.69	1.252
NCTa1-CeO_2	0.201	1.80	2.8751	14.2108	101.724	2.87	9.26	7.38	1.100

Table 4 The R_{sf} and R_{ct} data extracted from the Nyquist plots with respect to cycle number

Samples	Pristine NC		NCTa1		NCTa1-CeO_2	
	R_{sf} (Ω)	R_{ct} (Ω)	R_{sf} (Ω)	R_{ct} (Ω)	R_{sf} (Ω)	R_{ct} (Ω)
10 th	73.4	82.8	26.6	25.6	12.4	24.8
40 th	93.8	130.4	30.9	68.7	12.6	48.0
70 th	109.3	233.8	40.0	123.0	12.5	64.1
100 th	123.7	448	48.9	159.0	13.4	81.1

Table 5 Electrochemical performance of recent Ni-rich oxide materials reported in the literatures.

Modified strategy	Cutoff voltage (V)	Operating temperature (℃)	1st Discharge capacity at 0.1 C (mAh g^{-1})	Cell type for cycling	1st Discharge capacity for cycling (mAh g^{-1})	Cycling performance	Capacity loss per cycle	Reference
**NCTa1-CeO_2**	**2.7-4.5**	**25**	**231.9**	**Full-cell (2.7-4.5V)**	**204.7 (1C)**	**90.9% (1C, 1000 cycles)**	**0.0091%**	**This work**
Li_3PO_4-coated $LiNi_{0.76}Co_{0.1}Mn_{0.14}O_2$	2.7-4.5	25	220	Half-cell	~200 (0.3C)	91.6% (0.3C, 200 cycles)	0.0420%	12
PEDOT-coated $LiNi_{0.85}Co_{0.1}Mn_{0.05}O_2$	2.7-4.3	25	~210	Half-cell	177 (1C)	91% (1C, 100 cycles)	0.0900%	13
Co/B-coated $LiNi_{0.8}Co_{0.1}Mn_{0.1}O_2$	3.0-4.4	25	214	Full-cell (2.7-4.3V)	/	95% (1C, 500cycles)	0.0100%	14
Ta-doped $LiNi_{0.9}Co_{0.1}O_2$	2.7-4.3	30	229	Full-cell (3.0-4.2 V)	~190 (1C)	90% (1C, 2000cycles)	0.0050%	15
LYTP-coated	2.75-4.4	25	216.6	Full-cell	200 (0.5C)	85% (1C,	0.0150%	16

$LiNi_{0.88}Co_{0.09}Mn_{0.03}O_2$				(2.75-4.4 V)		1000cycles)		
Gradient designed $LiNi_{0.8}Co_{0.1}Mn_{0.1}O_2$	2.8-4.4	25	208	Half-cell	~195 (0.5C)	92% (0.5C, 100cycles)	0.0800%	17
Graphene-coated $LiNi_{0.8}Co_{0.15}Al_{0.05}O_2$	2.75-4.3	30	~205	Full-cell (2.75-4.2V)	/	70% (0.5C, 300cycles)	0.1000%	18
SO_2-treated $LiNi_{0.91}Co_{0.06}Mn_{0.03}O_2$	3.0-4.3	25	216.1	Half-cell	180 (1C)	91% (1C, 100 cycles)	0.0900%	19
Ta-doped $LiNi_{0.8}Co_{0.1}Mn_{0.1}O_2$	3.0-4.3	25	211.2	Half-cell	~190 (0.5C)	90.4% (0.5C, 100cycles)	0.096%	20
Al/Mg-doped $LiNi_{0.9}Co_{0.05}Mn_{0.05}O_2$	2.8-4.4	25	213	Full-cell (2.5-4.2 V)	~170 (1C)	84% (1C, 1000cycles)	0.0160%	21
W-doped $LiNi_{0.9}Co_{0.05}Mn_{0.05}O_2$	2.7-4.3	30	~225	Full-cell (3.0-4.2 V)	~183 (1C)	~89% (1C, 1000cycles)	0.0110%	22
Nb-doped $LiNi_{0.8}Co_{0.1}Mn_{0.1}O_2$	2.8-4.6	25	225.1	Half-cell (2.8-4.4 V)	~180 (1C)	93.2% (1/3C, 250 cycles)	0.0272%	23
PMMA-coated $LiNi_{0.8}Co_{0.1}Mn_{0.1}O_2$	2.8-4.3	25	206.1	Half-cell	181.1 (1C)	91.2% (1C, 100 cycles)	0.0880%	24
B-doped $LiNi_{0.9}Co_{0.1}O_2$	2.7-4.3	30	234	Half-cell	~220 (0.5C)	91.2% (1C, 100 cycles)	0.0880%	25
Co-coated $LiNi_{0.91}Mn_{0.03}Co_{0.06}O_2$	3.0-4.35	30	225	Full-cell (2.7-4.25V)	~202 (0.5C)	85% (1C, 300 cycles)	0.0500%	26
PP10-Li-coated $LiNi_{0.8}Co_{0.1}Mn_{0.1}O_2$	3.0-4.3	20	203	Half-cell	180 (1C)	81.1% (1C, 500 cycles)	0.0378%	27
Ti-doped/La coated $LiNi_{0.8}Co_{0.1}Mn_{0.1}O_2$	2.7-4.3	25	200.4	Half-cell	174.82 (1C)	90.6% (1C, 200 cycles)	0.0470%	28
Mg-doped $LiNi_{0.94}Co_{0.06}O_2$	2.8-4.4	25	214	Half-cell	~200 (1/3C)	90% (0.3C, 150 cycles)	0.0667%	29

Table 6 Structural parameters of NCTa1-CeO_2 and pristine NC from the EXAFS fitting.

Sample	State	Shell	N^a	R^b	σ^2 (10^{-3} $Å^2$)^d	ΔE_0 (eV)^c	R-factor^e
NCTa1-CeO_2	OCV	Ni-O	5.3 ±0.4	1.94 ±0.01	4.6 ±0.3	9.7 ±0.6	0.009
		Ni-M	6^f	2.87 ±0.01	7.2 ±0.2		
	Ch-4.5 V	Ni-O	5.1 ±0.3	1.89 ±0.01	4.2 ±0.7	10.0 ±0.6	0.009
		Ni-M	6^f	2.83 ±0.01	5.1 ±0.4		

	Disch-2.7 V	Ni-O	5.3±0.4	1.94±0.01	4.7±0.4	10.1±0.7	0.010
		Ni-M	6[f]	2.87±0.01	7.2±0.2		
Pristine NC	OCV	Ni-O	5.3±0.4	1.94±0.01	5.9±1.7	10.2±0.8	0.007
		Ni-M	6[f]	2.87±0.01	3.9±0.4		
	Ch-4.5 V	Ni-O	4.9±0.4	1.89±0.01	4.3±1.5	9.8±0.4	0.010
		Ni-M	6[f]	2.83±0.01	6.7±0.7		
	Disch-2.7 V	Ni-O	4.9±0.5	1.94±0.01	6.8±1.9	10.0±0.4	0.008
		Ni-M	6[f]	2.87±0.01	5.1±0.5		

[a]N is coordination number; [b]R is the internal atomic distance (the bond length between Ni central atoms and surrounding coordination atoms; [c]ΔE_0 is the edge-energy shift (the difference between the zero kinetic energy value of the sample and that of the theoretical model); [d]σ^2 is Debye-Waller factor (represents the thermal and static disorder in absorber-scatterer distances); [e]R-factor represents the fitness of data processing; [f]These coordination numbers were constrained as N(Ni-M) = 6.

Table 7 The fitting results of Ce *L*-edge XANES spectra.

State	Center (eV)	σ	Area	R-factor	χ^2
OCV	5725.9	4.454	7.230	0.003	0.498
	5730.0	2.429	7.506		
	5737.0	3.325	10.433		
Ch-4.5 V	5725.9	3.103	3.543	0.001	0.059
	5730.0	2.639	6.625		
	5737.0	3.278	7.354		
Disch-2.7 V	5725.9	3.750	8.915	0.002	0.613
	5730.0	2.348	9.258		
	5737.0	3.269	13.440		

Notes: The adsorption peak at 5725.9 eV is ascribed to Ce^{3+}, which corresponds to the electron transition from $2p_{3/2}$ to $(4f^1)5d$. Two distinct peaks at 5730.0 and 5737.0 eV are derived from Ce^{4+}, which correspond to the transition from $2p^{3/2}$ to $(4f_L)5d$ and $(4f^0)5d$, respectively[30]. The L denotes that an electron in 2p orbital of oxygen is transferred to 4f orbital of Ce. Therefore, according to the area ratios of Gaussian function for Ce^{3+} and Ce^{4+}, the average valence of Ce ions can be obtained.

Reference

[1] Hafner, J. Ab-initio simulations of materials using VASP: Density-functional theory and beyond. *J. Comp. Chem.* **29**, 2044-2078 (2008).

[2] Perdew, J. P., Burke, K. & Ernzerhof, M. Generalized gradient approximation made simple. *Phys. Rev. Lett.* **77**, 3865-3868 (1996).

[3] Kresse, G. & Joubert, D. From ultrasoft pseudopotentials to the projector augmented-wave method. *Phys. Rev. B* **59**, 1758-1775 (1999).

[4] Becke, A. D. Density‐functional thermochemistry. IV. A new dynamical correlation functional and implications for exact‐exchange mixing. *J. Chem. Phys.* **104**, 1040-1046 (1996).

[5] Grimme, S., Antony, J., Ehrlich, S. & Krieg, H. A consistent and accurate ab initio parametrization of density functional dispersion correction (DFT-D) for the 94 elements H-Pu. *J. Chem. Phys.* **132**, 154104 (2010).

[6] Blöchl, P. E. Projector augmented-wave method. *Phys. Rev. B* **50**, 17953-17979 (1994).

[7] Li, M. et al. XPS analyses on Ta/Au/NiFe/NiO/Ta films. *Surf. Interface Anal.* **47**, 540-544

(2015).

[8] Yu, H., Li, Y., Hu, Y., Jiang, H. & Li, C. Concurrently coating and doping high-valence vanadium in nickel-rich lithiated oxides for high-rate and stable lithium-ion batteries. *Ind. Eng. Chem. Res.* **58**, 4108-4115 (2019).

[9] Li, Y. et al. A novel electrolyte salt additive for lithium-ion batteries with voltages greater than 4.7 V. *Adv. Energy Mater.* **7**, 1601397 (2017).

[10] Xu, M. et al. Development of Novel Lithium Borate Additives for Designed Surface Modification of High Voltage ($LiNi_{0.5}Mn_{1.5}O_4$) Cathode. *Energy Environ. Sci.* **9**, 1308-1319 (2016).

[11] Sasaki, T. et al. Capacity-fading mechanisms of $LiNiO_2$-based lithium-ion batteries: I. Analysis by electrochemical and spectroscopic examination. *J. Electrochem. Soc.* **156**, A289-A293 (2009).

[12] Yan, P. et al. Tailoring grain boundary structures and chemistry of Ni-rich layered cathodes for enhanced cycle stability of lithium-ion batteries. *Nat. Energy* **3,** 600–605 (2018).

[13] Xu, G. L. et al. Building ultraconformal protective layers on both secondary and primary particles of layered lithium transition metal oxide cathodes. *Nat. Energy* **4,** 484–494 (2019).

[14] Yoon, M. et al. Reactive boride infusion stabilizes Ni-rich cathodes for lithium-ion batteries. *Nat. Energy* **6,** 362–371 (2021).

[15] Kim, U. H. et al. Heuristic solution for achieving long-term cycle stability for Ni-rich layered cathodes at full depth of discharge. *Nat. Energy* **5,** 860–869 (2020).

[16] Fan, X. et al. In situ inorganic conductive network formation in high-voltage single-crystal Ni-rich cathodes. *Nat. Commun.* **12,** 5320 (2021).

[17] Liu, T. et al. Rational design of mechanically robust Ni-rich cathode materials via concentration gradient strategy. *Nat. Commun.* **12,** 6024 (2021).

[18] Park, C.W. et al. Graphene collage on Ni-rich layered oxide cathodes for advanced lithium-ion batteries. *Nat. Commun.* **12,** 2145 (2021).

[19] Seong, W. M.et al. Controlling residual lithium in high‐nickel (> 90%) lithium layered oxides for cathodes in lithium‐ion batteries. *Angew. Chem. Int. Edit.* **59**, 18662-18669 (2020).

[20] Zou, Y. G. et al. Mitigating the kinetic hindrance of single‐crystalline Ni‐rich cathode via surface gradient penetration of tantalum. *Angew. Chem. Int. Edit.* **133**, 26739-26743 (2021).

[21] Li, W., Lee, S. & Manthiram, A. High‐nickel NMA: a cobalt‐free alternative to NMC and NCA cathodes for lithium‐ion batteries. *Adv. Mater.* **32**, 2002718 (2020).

[22] Kim, U. H. et al. Pushing the limit of layered transition metal oxide cathodes for high-energy density rechargeable Li ion batteries. *Energy Environ. Sci.* **11**, 1271-1279 (2018).

[23] Xin, F. et al. What is the role of Nb in nickel-rich layered oxide cathodes for lithium-ion batteries?. *ACS Energy Lett.* **6**, 1377-1382 (2021).

[24] Han, Y., Heng, S., Wang, Y., Qu, Q. & Zheng, H. Anchoring interfacial nickel cations on single-crystal LiNi0. 8Co0. 1Mn0. 1O2 cathode surface via controllable electron transfer. *ACS Energy Lett.* **5**, 2421-2433 (2020).

[25] Ryu, H. H., Park, N. Y., Yoon, D. R., Kim, U. H., Yoon, C. S. & Sun, Y. K. New class of Ni‐rich cathode materials $Li[Ni_xCo_yB_{1-x-y}]O_2$ for next lithium batteries. *Adv. Energy Mater.* **10**, 2000495 (2020).

[26] Kim, Y., Park, H., Shin, K., Henkelman, G., Warner, J. H. & Manthiram, A. Rational design of coating ions via advantageous surface reconstruction in high‐nickel layered oxide cathodes for lithium‐ion batteries. *Adv. Energy Mater.* **11**, 2101112 (2021).

[27] Chen, Z. et al. Lithium phosphonate functionalized polymer coating for high‐energy Li $[Ni_{0.8}Co_{0.1}Mn_{0.1}]O_2$ with superior performance at ambient and elevated temperatures. *Adv. Funct. Mater.* **31**, 2105343 (2021).

[28] Yang, H.et al. Simultaneously dual modification of Ni‐rich layered oxide cathode for high‐energy lithium‐ion batteries. *Adv. Funct. Mater.* **29**, 1808825 (2019).

[29] Xie, Q., Li, W. & Manthiram, A. A Mg-doped high-nickel layered oxide cathode enabling safer, high-energy-density Li-ion batteries. *Chem. Mater.* **31**, 938-946 (2019).

[30] Takahashi, Y., Sakami, H. & Nomura, M. Determination of the oxidation state of cerium in rocks by Ce L_{III}-edge X-ray absorption near-edge structure spectroscopy. Anal. Chim. Acta **468**, 345–354 (2002).
